# Hepatitis B Virus-Associated Hepatocellular Carcinoma

**DOI:** 10.3390/v14050986

**Published:** 2022-05-07

**Authors:** Giacomo Emanuele Maria Rizzo, Giuseppe Cabibbo, Antonio Craxì

**Affiliations:** 1 Section of Gastroenterology & Hepatology, Department of Health Promotion, Mother and Child Care, Internal Medicine and Medical Specialties, PROMISE, University of Palermo, 90127 Palermo, Italy; g.rizzo.gr@gmail.com (G.E.M.R.); antonio.craxi@unipa.it (A.C.); 2 Department of Surgical, Oncological and Oral Sciences (Di.Chir.On.S.), University of Palermo, 90127 Palermo, Italy; 3 Endoscopy Service, Department of Diagnostic and Therapeutic Services, IRCCS-ISMETT, 90127 Palermo, Italy

**Keywords:** hepatitis B virus, hepatitis B, hepatitis B chronic hepatitis, hepatocellular carcinoma, hepatitis D virus

## Abstract

Hepatitis B virus (HBV) is DNA-based virus, member of the Hepadnaviridae family, which can cause liver disease and increased risk of hepatocellular carcinoma (HCC) in infected individuals, replicating within the hepatocytes and interacting with several cellular proteins. Chronic hepatitis B can progressively lead to liver cirrhosis, which is an independent risk factor for HCC. Complications as liver decompensation or HCC impact the survival of HBV patients and concurrent HDV infection worsens the disease. The available data provide evidence that HBV infection is associated with the risk of developing HCC with or without an underlying liver cirrhosis, due to various direct and indirect mechanisms promoting hepatocarcinogenesis. The molecular profile of HBV-HCC is extensively and continuously under study, and it is the result of altered molecular pathways, which modify the microenvironment and lead to DNA damage. HBV produces the protein HBx, which has a central role in the oncogenetic process. Furthermore, the molecular profile of HBV-HCC was recently discerned from that of HDV-HCC, despite the obligatory dependence of HDV on HBV. Proper management of the underlying HBV-related liver disease is fundamental, including HCC surveillance, viral suppression, and application of adequate predictive models. When HBV-HCC occurs, liver function and HCC characteristics guide the physician among treatment strategies but always considering the viral etiology in the treatment choice.

## 1. Introduction

Liver cancer is one of the most common malignancies worldwide, with approximately 840,000 new cases (its incidence ranks third among all cancers) and 780,000 deaths reported in 2018, ranking as the third mortality among all cancers [1]. Hepatocellular carcinoma (HCC) accounts for 90% of primary liver cancer cases, so it is considered a challenging public health issue. The prevalence of HCC in the US and in Western Europe is increasing, with most patients with HCC having an underlying cirrhosis, which was mainly secondary to hepatitis B virus (HBV) or hepatitis C virus (HCV) infection in the past [2]. In Italy, 10,000 cases of cancers yearly are pathogen-related, with 34.7% of them attributable to liver cancer of viral etiology (HBV- and HCV-related) [3]. However, the incidence of HCC related to viral liver disease is decreasing in developed countries, and it is counterbalanced by an increasing prevalence of nonalcoholic fatty liver disease (NAFLD) and its related HCC [4]. Unfortunately, a great number of HCC cases is still diagnosed in an advanced stage, and this generally restricts the efficacy of therapies. Moreover, liver cirrhosis is the strongest risk factor for HCC, so its decompensation together with tumour recurrence contribute to long-term mortality, even when curative treatment of early HCC is achieved. The prognosis of HCC patients, indeed, is relatively poor compared with other gastrointestinal (GI) tract tumors, since the 5-year survival rate is less than 20% [5]. However, HBV is the major risk factor of liver cirrhosis in eastern countries, where it has its higher prevalence and incidence, and so HBV-related HCC has consequently a higher incidence in Asia [6]. Specifically, the prevalence of HBV infection in the general Chinese population was approximately 5–7.99% in 2018, of which more than 90% were adults older than 20 years. This means that even if prevention and vaccination programs decreased infections over the past 10 years, at least an estimated population of 84 million is still infected, which is more than in any country of Europe [7].

Regarding its structure, HBV is a DNA-based virus and a member of the Hepadnaviridae family, which increases the risk of HCC in infected individuals through different mechanisms. Viral transmission may be sexual or occur through an exposure to infect blood/bodily fluids, with the majority of exposures currently occurring from mother to child through vertical transmission during birth or early years of life. Fortunately, universal childhood HBV vaccination has been implemented worldwide since 1992 and reduced 90% of chronic hepatitis B (CHB) prevalence in the vaccinated cohorts, as shown in Asian countries [8,9], resulting in a parallel decline of young-age HCC [10]. Despite the implementation of vaccination programs, the World Health Organization (WHO) reports that 296 million individuals were living with CHB infection in 2019, with 1.5 million new infections every year. Moreover, among those CHB individuals, about 60 million are co-infected with hepatitis D virus (HDV) [11]. Actually, the true prevalence of HDV co-infected HBV patients worldwide still remains unknown due to its heterogeneity, varying from 15–20 to 72 million [12,13]. Those individuals who are HBV–HDV coinfected had no therapies available for both viruses so far, but new treatment strategies have been recently developed [14]. Although there are treatment options to lower the risk of HCC in those who have chronic HBV infection, such as nucleos(t)ide analogs [15], globally many individuals are unaware of their status and lack access to testing and curative treatment.

In this review article, we discuss the relationships between HBV and HCC in terms of viral oncogenic mechanisms involving HBV-pathways, current best treatment options in HBV-related HCC, the burden of concomitant HDV infection and the impact of HBV-related chronic liver disease are the outcomes of HCC treatments.

## 2. Literature Review and Search Criteria

Studies for this review were retrieved from PubMed using the search terms “hepatocellular carcinoma”, “liver cancer”, and “primary liver carcinoma”, both individually and in combination with the terms “hepatitis B virus”, “HBV”, “HDV”, “chronic hepatitis”, “cirrhosis”, “liver function”, “antivirals”, “HBx”, “molecular pattern”, “NUC”, “nucleoside analogs”, “nucleotide analogs”, as well as by a manual search and review of reference lists. The search included literature published in English until March 2022. The authors independently evaluated and selected the studies retrieved and resolved any discrepancies by discussion. All authors approved the final list of selected articles.

## 3. Epidemiology of Hepatitis B Virus Infection and Liver Cancer

In Italy, the prevalence of HBsAg carriers in the general population decreased from 3% in the 1980s to 1% in 2010 [16]. In 2019, a national Italian study recruited 894 CHB patients, of whom 783 were tested for Anti-HCV, showing that 3.1% of them had co-existing HCV or HBV infection [17]. In 2018, Valery et al. reported a projected increase in liver cancer incidence to 2030 in 30 countries. In the interval between 1978 and 2012, HCC incidence declined in many eastern countries, even as it increased in India, Oceania, and most western countries. Italy was analyzed through a regional population-based cancer registry including nine regions, which surely limited the results but also showed a projection of new cases yearly of liver cancer from approximately 7036 in 2005 to about 10,642 in 2030 [18]. Recently, however, the increase in some countries, such as the US, has abated as a consequence of having reached a plateau in various subgroups [19]. Overall, the lifetime incidence of HCC in HBV individuals has been reported to be approximately 10–25% [20]. Furthermore, other major hepatotropic viruses also increase the risk of HCC development, both coinfection with hepatitis D virus (HDV) and the presence of HCV. Previously, HDV was shown to infect about 5% of CHB patients worldwide (15–20 million individuals) [21], while recently the overall prevalence of HDV seemed to be 0.98%, with a prevalence of HDV in CHB reaching 10.6%, which is twice as high as previous estimations [13]. Consequently, there are considerable differences in these virus-induced HCC populations, mainly regarding the epidemiology of HBV–HDV-associated HCC, which is more common in developing countries, while HCV-induced HCC is more common in post-industrial society [22]. In comparison to HBV or HCV mono-infection, individuals who are co-infected with HBV–HCV have increased rates of HCC development [23]. In general, risk factors for HCC in both treated and untreated CHB patients may be demographic (male sex, older age, Asian or African ancestry), viral (high viral load, long infection, coinfection with HCV, human immunodeficiency virus, or HDV), clinical (liver cirrhosis), and environmental (aflatoxin, alcohol, or tobacco) factors [24].

## 4. Structure and Replication Cycle of Hepatitis B Virus

HBV is an enveloped, double-stranded DNA virus belonging to the *Orthohepadnavirus* genus and the Hepadnaviridae family, and its classification includes 10 genotypes (A–J) [25]. The HBV virion contains 3020 to 3320 nucleotides in a partially double-stranded DNA genome (in the form of relaxed circular DNA, rcDNA) in a nucleocapsid composed of hepatitis B core antigen (HBcAg) subunits. This nucleocapsid is enveloped in a host-derived lipid bilayer covered with hepatitis B surface antigens (HBsAg). The genome contains four genes (P, preC/C, S and X) that encode for five main proteins: polymerase (gene P), HBcAg (gene C), hepatitis B envelope antigen (HBeAg) (product of preC), HBsAg (gene S), and a replication cofactor X (gene X) [26,27]. The X protein (HBx) derived from gene X plays an essential role in HBV pathogenesis and viral transcription, and nowadays its role in carcinogenesis of HCC is recognized to be relevant, as is analyzed below [27,28,29]. On a hepatocyte level, the infection begins with the attachment of the virion to the sodium taurocholate co-transporting polypeptide (NTCP), the entry receptor of the virus [30,31]. The virus uses host cell DNA repair enzymes to successfully convert HBV rcDNA into the HBV covalently closed circular DNA (cccDNA) form, which is an episomal “mini-chromosome” when associated with histone and non-histone proteins and acts as a stable template for five viral mRNAs [32,33]. These mRNAs include the pregenomic RNA (pgRNA), the precore RNA, the surface mRNAs, and the X mRNA. The synthesis of rcDNA (the major pathway) or double-stranded linear DNA (dslDNA) results through a reverse transcription process in the HBV nucleocapsid, which matures and becomes enveloped by HBsAg and secreted into the blood at multi-vesicular bodies [34]. Conversely to rcDNA, nuclear dslDNA genomes can form replication-defective cccDNA [35] or integrate into the host cell genome [36,37]. A broad range of components generated by virus-infected cells has been investigated as potential biomarkers for predicting HCC occurrence and recurrence.

### 4.1. HBV DNA Integration

HBV uses reverse transcription for replication, but integration is not essential in the virus lifecycle because it does not produce replication-competent virus [38]. During reverse transcription of the pgRNA, partially double-stranded rcDNA is formed 90% of the time, and dslDNA is synthesized for the residual 10% of cases [37]. The HBV dslDNA can also be present in virions and repaired to produce cccDNA [36]. Integration of dslDNA is an event reported to occur in 1 of approximately 10,000,000 infected hepatocytes, and populations with higher percentage of dslDNA integration are children (as young as 5 months old) and patients who have acute HBV, CHB, and HCC [39,40]. Tu et al., in a review in 2017, proposed the current and accepted mechanisms for HBV integration driving HCC, which include: (1) chromosomal instability from HBV integrated DNA; (2) insertional mutagenesis in proto-oncogenes and tumor suppressors; and (3) expression of mutant HBV proteins [37]. Regarding the first mechanism, non-HCC patients showed integration sites randomly distributed through the genome but lacking in enriched sites, causing alterations, while HBV integration in CHB-HCC patients is enriched in certain areas causing chromosomal instability. In the latter cases, integration usually occurs near fragile sites such as intergenic regions, CpG islands, simple repeats, repetitive regions, and telomeres, bringing us to the second mechanism, inducing HCC [41]. As confirmation, next-generation sequencing (NGS) studies have found that HCC tumors generally have a greater number of integration events and increased integration frequency in coding or promoter regions when comparing HBV integration sites between tumor and matched non-tumor tissues [37]. In 10–15% of HCC cases there is an upregulation of oncogenes of the enhancer II/core HBV promoter in/near telomerase reverse transcriptase (TERT) or myeloid/lymphoid or mixed-lineage leukemia 4 genes caused by a recurrent integration of dslDNA [42,43]. Due to the evidence of upregulation of these genes in early and late HCC development, it is supposed that integration in these genes may be linked to both cell transformation and HCC progression. Moreover, integration of dslDNA generates high expression of altered HBsAg and HBx proteins, which is associated with endoplasmic reticulum (ER) and mitochondrial stress responses, which may lead to the increased risk of HCC [44]. In fact, past studies in animal models showed over-expression of mutant HBsAg and HBx into precancerous liver lesions and HCC [45]. Moreover, expression of C-terminal truncated HBx protein from integrated HBV is associated with inhibition of apoptosis and cell transformation [46].

### 4.2. HBx Protein and Its Role

**Figure 1:**
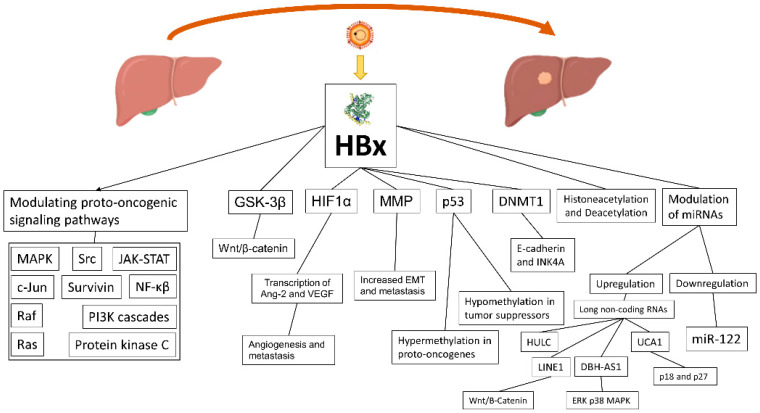
The role of HBx on HCC development in HBV-related HCC.

The HBx protein weights 17 kDa and has no direct interaction with the genome, but nonetheless it performs different roles in the HBV lifecycle and HCC development (Figure 1) [47]. HBx localizes in the cytoplasm, nucleus, and mitochondria, where it affects signal transduction, transcription, and mitochondrial function. Consequently, it causes transactivation of viral and cellular genes and leading to four main mechanisms contributing to HCC development: (1) integration of the HBx gene into the hepatocyte genome, which promotes genetic instability; (2) induction of oxidative stress through the interaction with the mitochondrial and other cellular proteins; (3) activation of cell survival signaling pathways and inactivation of tumor-suppressors; and (4) induction of epigenetic modifications such as histone acetylation, DNA methylation, and microRNA expression [48]. As a consequence, HBx is able to modulate many proto-oncogenic signaling pathways involved in inflammation and proliferation, such as mitogen-activated protein kinase (MAPK)/Ras/Raf/c-Jun, NF-κβ, JAK-STAT, protein kinase C, Src, survivin, and PI3K cascades [49,50]. The Wnt/β-catenin pathway, another relevant oncogenic pathway, seems to be activated by HBx, through binding antigen-presenting cell protein or inactivation of GSK-3β through extracellular signal regulated kinase activation. The result is the accumulation of β-catenin and an increased transcription of pro-angiogenic/metastatic factors [51,52]. Promotion of angiogenesis and metastasis is mediated by transcription of Ang-2 and vascular endothelial growth factor (VEGF), which is activated by HIF1α, a factor whose transcriptional activation/stabilization is promoted by hypoxic cirrhotic nodules expressing HBx, resulting in increased survival and growth [53]. HBx also modulates matrix metallo proteinases (MMP), which digest fibrous capsules in tumors with the result of increasing the epithelial–mesenchymal transition (EMT) and metastasis [54]. HBx may also bind p53 in the cytoplasm and prevent p53 nuclear localization, with loss of p53 activity, genome instability, and the deregulation of tumor suppressors [55]. Furthermore, HBx is an epigenetic regulator of DNA hypermethylation in proto-oncogenes and hypomethylation in tumor suppressors [56]. Another mechanism of HBx in tumour genesis is through alteration of the transcription process of the methyl catalase DNMT1, which hypermethylates, when upregulated, the tumor suppressor gene E-cadherin and INK4A, resulting in loss of cell-cycle regulation and promoting EMT [57]. Promotion of histone acetylation and deacetylation is another mechanism of HBx to alter the expression of cancer-related genes, microRNAs, and non-coding RNAs. Several miRNAs are downregulated by HBx, and among them is miR-122, a liver-specific miRNA with an anti-tumorigenic role [58]. Quite the opposite, but with the same result, HBx induces expression of long non-coding RNAs: LINE1, which upregulates Wnt/B-Catenin, HULC, UCA1 (with inhibition of tumor suppressors p18 and p27), and DBH-AS1 (activation of extracellular signal-regulated kinase [ERK]/p38/MAPK) [59].

### 4.3. Intracellular Oxidative Stress in HBV-Infected Hepatocytes

Oxidative stress results from a higher presence of elements or reactions as lipid peroxidation, 8-oxoguanine DNA products, and decreased levels of anti-oxidant enzyme glutathione and oxidation of proteins. CHB patients have 1.5–4 times higher levels of these findings in the liver and blood compared to HBV-negative individuals [60]. Furthermore, HBx has a main role in increasing the oxidative stress, firstly interacting with cytochrome c oxidase subunit 3 (COXIII), a protein related to mitochondrial respiratory chains, causing an increase in mitochondrial reactive oxygen species (mitoROS) levels, which results in mitochondrial dysfunction [61]. Analysis of HCC tissue of HBV patients showed that HBx activated NLRP3 in normal hepatocytes under conditions of oxidative stress and promoted pyroptosis via the mitoROS pathway, causing an increase of levels of ASC, IL-1β, IL-18, and HMGB1 [62]. Moreover, HBx also localizes on the outer mitochondrial membrane, as abovementioned, causing reduced expression and activity of respiratory complex proteins I, II, IV, and V in the electron transport. As a consequence, cellular respiration reduces, and the altered mitochondrial function increases production of superoxide anions and 8-oxoguanine DNA [63,64]. During the HBV lifecycle, HBsAg and HBeAg are folded and assembled in the ER and transported through the Golgi [65]. When high levels of these proteins or mutant HBV proteins are misfolded, they can accumulate in the ER and cause activation of an unfolded protein response (UPR), which causes release of hydrogen peroxide and calcium ions into the cytoplasm, enhancing ROS production [66]. Furthermore, mutations in HBcAg in CHB genotype C individuals seem able to increase ER stress, resulting in ROS, increased intracellular calcium (Ca^2+^), and higher level of proinflammatory cytokines [67]. HBV infection also decrease antioxidative stress response pathways, with the result of concurrently increasing total oxidative stress [68].

## 5. HBV Oncogenic Mechanisms: What We Know from Literature

HBV possesses different associated mechanisms to promote tumorigenesis, specifically through the activation and/or deactivation of various pathways, causing HCC [69]. Moreover, HBV has the unique finding among the hepatotropic viruses of being able to generate HCC in the absence of cirrhosis, although most cases of HBV-related HCC (about 70–90%) occur in cirrhotic patients [70]. Several steps of the viral and hepatocyte life cycle are directly and indirectly involved, as much as the alteration of the microenvironment homeostasis. Several mutations of the HBV genome are associated with a higher risk of HCC, and they can affect any of the HBV genes (PreS/S, P, PreC/C, X) [71]. Therefore, studies including review and meta-analysis explored the role of these mutations, and the resulting HBV mutants showed differences in the frequency between HCC vs. non-HCC patients, with a risk of HCC ranging from 1.83- to 5.34-fold [72,73,74,75,76,77]. In addition, a proteogenomic characterization of HBV-related HCC using paired tumor and adjacent liver tissues from 159 Chinese patients provided a comprehensive and integrated analysis. Among the 159 HBV-HCCs, five significantly mutated genes were identified, including TP53 (58%), CTNNB1 (19%), AXIN1 (18%), KEAP1 (7%), and RB1 (6%), two of which (CTNNB1 and AXIN1) were mutually exclusive. Furthermore, this study identified PYCR2 (a crucial enzyme in proline biosynthesis) and ADH1A (enzyme metabolizing a wide variety of zenobiotic compounds) as prognostic biomarkers (confirmed by multivariate analysis: PYCR2 high versus low, HR 1.792; ADH1A low versus high, HR 2.703) involved in HCC metabolic reprogramming [78]. Moreover, other genes including TERT (its resulting protein is a subunit of telomerase and maintains genomic integrity) and RPS6KA3 hold frequently somatic mutations in HBV-associated HCC [79,80,81], while alterations in genes such as ARID1A and ARID2 (chromatin regulator genes, encoding for chromatin remodeling factors) can cause epigenetic modifications leading to HCC development in these patients [82]. Actually, even if HBV-associated HCCs hold the latter mutations, they are more frequent in HCV-associated HCC (TERT, 60–80% in HCV-HCC vs. 30–40% in HBV-HCC [83,84,85]; ARID2, 18% in HCV-HCC vs. 2% in HBV-HCC [86]).

## 6. Immune System and Microenvironment in HBV–HCC

The immunosuppressive activity of regulatory T cells (TREG) and their role in tumour progression has been well documented in different cancers, including HCC [87,88]. CD8+ resident memory T cells (TRM) were suggested to show a partial immune response in chronic HBV infection [89]. In 2019, Tim et al. showed that TREG and TRM were enriched in the tumour microenvironment (TME) of HBV-related HCC through a transcriptome analysis. According to their results, TREG from HBV-related HCC showed higher expression of FOXP3 (as already known [90]) and other genes involved in the IL-10 pathways, indicating a more immunosuppressive phenotype of TREG compared with non-viral-related HCC. On the other hand, genes enriched in TRM (CD8+CD103+) from HBV-related HCC exhibited a state of exhaustion, as demonstrated by the higher expression of exhaustion marker genes such as PD-1, LAG3, HAVCR2 (Tim-3), and CTLA4. Survival analysis showed a poorer overall survival (OS) in HBV-related HCC with high levels of tumour-infiltrating TREG, whereas a higher number of tumour-infiltrating TRM was associated with a better survival profile. Authors further validated that the TME of HBV-related HCC is more immunosuppressive/exhausted than non-viral HCC [91]. Moreover, the expression of PD-1 is increased on these TREG and TRM, supposing to have a higher response of HBV-related HCC to immunotherapy as PD-1 inhibitors [92].

## 7. Clinical Aspect of HBV Infection and Its Progression to HCC

HBV patients may present various clinical manifestations, moving from an asymptomatic infection and arriving at acute liver disease with jaundice and liver failure. Moreover, this virus gives chronic infection more commonly in children than in adult; in fact, they are usually in the high-replication, low-inflammation phase of infection. Furthermore, liver cirrhosis and hepatocellular carcinoma are rare in children [93]. When infection arises that is chronic and asymptomatic, it may be unacknowledged for years, despite the production of high levels of virus antigens and viral particles by the liver. Nonetheless, HBV can trigger an immune response after decades of infection, but it is generally insufficient to clear all HBV-infected liver cells, causing subsequently chronic inflammation and liver damage progression. The European clinical guidelines on chronic HBV infection [94] classified patients at presentation into four classes (which corresponds to the four phases of HBV natural history) depending on the identification of the virological markers (HBsAg and HBeAg), viral load (HBV-DNA), and the seroconversion of antibodies against HBeAg (Anti-HBe antibodies). In general, CHB leads to a repeated cycle of liver damage and regeneration, which promotes tumorigenesis [95]. The ultimate step of the progression of the HBV-related liver disease is liver cirrhosis, which is undoubtedly the main risk factor for HCC development. Antiviral therapies aim to suppress viral replication, whose suppression interrupts liver disease progression and decreases risk of cirrhosis complications, such as decompensation and HCC.

## 8. Impact of HDV Infection on Liver Disease and HCC Development

HDV is a hybrid and defective virus of a 36 nm particle, containing a single-stranded circular RNA genome of about 1.7 Kb, and needs HBV to infect, so this unusual nature is also confounding in assigning a clear role in hepatocarcinogenesis [14]. Consequently, HDV can be disseminated from the individuals who HBV simultaneously infects (i.e., superinfection). Over 90% of HBV-superinfected individuals develop a chronic infection, and liver cirrhosis develops in 70% to 80% of the cases within 5 to 10 years after infection [96]. Liver HDV-related cirrhosis decompensates with an estimated annual incidence of 2.6–3.6%, and an incidence of HCC between 2.6% and 2.8%. HCC might be a consequence of the cumulative effect of both HBV and HDV, an effect of the underlying cirrhosis, or a direct oncogenic effect of HDV, but it is still unclear [97]. A meta-analysis showed a significantly increased risk of HCC in patients with chronic HDV hepatitis (CHD), with a pooled OR of 1.28 (95% CI 1.05–1.57; I2 = 67.0%), which increased to 2.77 in the absence of heterogeneity for prospective cohort studies (95% CI 1.79–4.28; I2 = 0%), compared to HBV monoinfection [98]. Nonetheless, data on the genomic signature of HDV or on the levels of HDV replication into the tumor are still lacking [99]. In a study conducted in Caucasian patients with HDV-HCC, gene expression was performed comparing malignant and non-malignant hepatocytes, reporting a molecular profile, which suggest that the molecular signature of HDV-HCC is different from HBV-HCC [100].

## 9. Surveillance and Scoring Predictive Systems for HBV Patients

HCC surveillance is fundamental to improving early detection, curative treatment receipt, and survival in patients with cirrhosis [101]. In general, HCC surveillance is based on transabdominal ultrasound (US) every 6 months, while the role of alpha-fetoprotein (AFP) is still a debated issue during surveillance, showing slight differences in the recommendations among the main guidelines (Table 1) [102,103]. In general, among patients with HBV infection, HCC surveillance is recommended for all patients with cirrhosis (with or without HBsAg seroclearance), or, among those without cirrhosis, depending on the presence of family history of HCC, age, and ethnicity. There is a certain grade of concordance among guidelines regarding the role of surveillance in cirrhotic HBV patients, while in non-cirrhotic HBsAg-positive patients, they show some difference in the recommendations. Americans guidelines suggest keeping surveillance on Asiatic HBsAg-positive males older than 40 years and females older than 50 years, and younger ages for Africans and African Americans (Africans with HBV could develop HCC at an age < 40 years) [104,105]. Europeans apply surveillance on HBsAg-positive patients at high risk of HCC after stratification according to scores and grade of fibrosis, while Asians have recommendation similar to those of Americans.

Several scoring systems were set up and also externally validated to predict the risk of HCC among CHB patients [107]. Despite their high negative predictive values (above 95%) for HCC occurrence over a 3- to 10-year period, some of them (CU-HCC, GAG-HCC, REACH-B, REACH-B II, LSM-HCC, RWS-HCC, D^2^AS RISK SCORE, HCC-ESC, and AGED) [108,109,110,111,112,113,114,115,116] were developed for untreated patients but were inadequate in patients receiving NUCs, which are the majority of the CHB cases nowadays. Regarding the latter setting of patients, many different scores have been developed in different populations [117,118,119,120,121,122,123], such as the CAMD score (cirrhosis, age, male sex, diabetes), which aimed to predict risk of HCC during the first few years of NUC treatment [124]. Previously, the PAGE-B model was developed and validated for Caucasian CHB patients receiving NUCs for the prediction of HCC development by 5 years [125,126], but only in 2018 was a modified PAGE-B score (including additional albumin levels) developed in eastern cohorts, showing to better predict the risk of HCC in CHB Asians under NUCs compared to the PAGE-B score [127]. In general, these models confirm that HCC incidence decreases with cumulative NA treatment, but data from a 10-center Caucasian cohort reported that a substantial risk of HCC remains beyond 5 years of entecavir or tenofovir treatment, especially in those patients with older age, lower platelets (both at baseline and year 5), and liver stiffness ≥ 12 kPa at year 5 [128]. These scores may have an important role in the identification of differences among intervals of surveillance, but no data in the literature indicate timings depending on them. Moreover, the feasibility of each of them in clinical practice depends on many factors, especially the ethnicity of the validation/derivation cohort, the staging of liver disease, and the antiviral treatment. We summarize the characteristics and the performances of the available scores in Table 2.

## 10. HBV Therapies and Risk of HCC Development

HCC prevention in the contest of CHB can be primary, secondary, and tertiary. Data on HBV treatments suggests that a significant amount of HCC cases could be avoided through secondary prevention [134]. Secondary prevention of HCC consists of the treatment of underlying liver diseases aiming at the prevention of disease progression [135]. The available therapies for HBV are mainly divided into two typologies: IFNs (interferons), and NUCs (nucleoside/nucleotide analogs). Data on the impact of IFN therapy (which includes IFN-α and pegylated IFN-α) in HBV-related cancers are contrasting, due to the lack of pre-treatment stratification for cancer predictors and exclusion of patients at higher risk of developing HCC, as those unfit for IFN due to advanced liver disease. IFN-α has the potential to target cccDNA, inducing the expression of ISG20 (interferon-stimulated gene product of 20 kDa) and activating apolipoprotein B editing complex 3 (APOBEC3) enzymes, which belong to APOBEC3A (A3A). Stadler et al. demonstrated that ISG20 is the nuclease responsible for an interferon-induced decline of cccDNA, and that the co-expression of catalytically active ISG20 and A3A can reduce cccDNA [136]. Unlike IFN-α, NUCs suppress viral replication but have no effect on HBV cccDNA, whose persistence plays a crucial role in chronic infection, inflammation, and cancer formation [137,138]. Nonetheless, the current international treatment guidelines for patients with CHB recommend NUCs due to their many benefits and considering the low response rate and poor safety of IFN-α. Therefore, entecavir (ETV), tenofovir disoproxil fumarate (TDF), and tenofovir alafenamide (TAF) are the first-line NUC treatments, and their indications have been expanded in the past two decades to cover considerably more CHB patient groups [94]. Oral NUCs are easier to prescribe and administer, and they also have higher safety compared to IFN and high efficacy in terms of viral suppression. Long-term ETV and TDF treatment resulted in decreased incidence rate of HCC [139,140]. In general, the risk of HCC occurrence is higher in those patients not achieving complete viral suppression, while HCC is better prevented in CHB rather than in cirrhotic patients when viral load is completely suppressed. In fact, viral load is found to be the most important factor leading to cirrhosis and its complications, including liver cancer development [141]. Kim et al. [142] found that in patients receiving NUCs, who with incomplete suppression, even with low levels of viremia (<2000 IU/mL), showed a higher risk to develop HCC compared to those with complete suppression (undetectable HBV-DNA). A re-analysis of outcomes after stratification for risk factors of HCC showed an association between NUC therapy and a reduced HCC risk only in younger non-cirrhotic patients, but it has to be considered that patients with cirrhosis have an intrinsically higher risk due to their advanced liver disease itself [143]. Among patients under antiviral treatments, cirrhosis, HBeAg-negative at baseline, and incomplete viral suppression were associated with an increased risk of HCC [144]. Immunologically, nucleotide analogues (TDF) were shown to induce higher serum interferon lambda-3 (IFN-k3) levels rather than nucleoside analogues (including lamivudine and ETV) [145]. IFN-k has antitumor activity in murine models with liver cancer [146], which may partly explain the difference in the lower HCC risk in TDF-treated patients. Even if ETV is effective to reduce incidence of HCC among CHB patients [147], the impact of TDF or ETV in different cohorts was analyzed, as was that in a Korean cohort of 1695 consecutive patients treated with ETV (*n* = 813) or TDF (*n* = 882) after curative-intent hepatectomy for HBV-related HCC. In this propensity score-matched analysis, the authors found TDF therapy to be associated with significantly higher recurrence-free (*p* = 0.02) and OS (*p* = 0.03) rates compared with ETV, and it resulted in an independent protective factor for both early (<2 years; HR 0.79) and late (≥2 years, HR 0.68) postoperative HCC recurrence [148]. These results were also confirmed in a meta-analysis of 15 studies (61,787 patients in total: 26.6% on TDF, and 73.94% on ETV), in which TDF treatment was associated with a significantly lower risk of HCC compared to ETV (HR 0.80; *p* = 0.003; I2 = 13%) [149]. Furthermore, age, cirrhosis, male sex, platelet count, liver stiffness, and diabetes are risk factors for HCC in CHB patients receiving NUCs [150]. Finally, the RECTRACT-B study showed that HBeAg-negative non-cirrhotic patients with low HBsAg levels are candidates for NUC withdrawal in order to increase chances to achieve HBsAg clearance (functional cure). These new insights in treatment strategy may expand worldwide indications for NUC interruption [151], but it is still unclear how the risk of HCC may change after NUC interruption.

## 11. Changing Perspective in HDV/HBV-Related HCC

As mentioned above, data suggest that both HDV-related liver disease and HBV–HDV-associated HCC may be more aggressive compared to those patients with monoinfection, showing also a higher rate of recurrence after treatment or LDLT (which will be discussed later). These outcomes were also the result of the lack of an effective therapy for reducing the burden of HDV so far, but new treatment options are now available with the potential to change the natural history and outcomes of these patients [152]. In July 2020, EMA (European Medicines Agency) approved the entry-inhibitor bulevirtide (BLV, previously named Myrcludex B) for the treatment of chronic HDV in HDV-RNA-positive patients with compensated liver disease, with a conditional marketing authorization after the encouraging results in small cohorts of CHD patients [153]. BLV could be used either in combination with peg-IFNα or as monotherapy, with differences in the dosage. Moreover, other drugs are under investigation, such as the prenylation inhibitor lonafarnib (LNF) or the nucleic acid polymer REP2139Ca (a molecular belonging to nucleic acid polymers, NAPs) [154,155]. Currently, two ongoing phase III studies are assessing the efficacy and safety of these new therapeutic regimens against HDV, namely MYR-301 (NCT03852719) for BLV, and D-LIVR (NCT03719313) for LNF. Surely, it is the beginning of the era of anti-HDV therapies, with important implications for the progression of liver disease and HCC development, so robust data with long-term follow up are needed in order to explore the new course of this disease.

## 12. General Indications for Treatment of HCC

HBV-related HCC does not follow different algorithms from the main worldwide indications, because of lack of evidence showing an HBV burden on influencing outcomes among different HCC treatments; indeed, the decision-making process is strongly dependent on the stage of tumor independently from the etiology of liver disease. In general, treatment strategies of HCC follow the Barcelona Clinic Liver Cancer (BCLC) [156] staging system, which was recently updated [157] and is based on disease burden presentation and underlying hepatic function, giving recommendations regarding the best therapeutic approach [158]. Liver function and performance status (according to the Eastern Cooperative Oncology Group (ECOG) staging system) are fundamental to stratify patients, proposing the best option according to their global status. In the early stage of cancer, curative therapies are available, and they are always more frequently able to control the progression of cancer thanks to serial interventions performed immediately when recurrence appears during follow up, resulting in a long OS. Depending on BCLC stage, initial curative options may include liver transplantation, resection, and/or ablation (radiofrequency ablation—RFA), while palliative or downstaging treatments may consider local–regional therapies (LRT), such as transarterial chemoembolization (TACE) or transarterial radioembolization (TARE), followed by systemic therapy in LRT-ineligible patients or those progressing on LRT [103,106]. Moreover, in those patients with unresectable hepatocellular carcinoma (uHCC) following TACE, the application of prognostic prediction models may help to choose the next management [159].

## 13. Outcomes of HBV-Related HCC Based on Treatment Choice

### 13.1. Treatment of Early HCC

A wise selection of candidates and applicability of curative therapies influence patient survival in terms of either early mortality due to liver decompensation or late mortality caused by tumor recurrence. Despite the role of HBV in tumorigenesis, generally its presence does not affect the decision-making process among treatments at the same HCC stage, because no treatment has had more efficacy in HBV-related HCC. In general, the 5-year recurrence rates of HCC change among curative treatments, moving from modest (4–18%) in selected patients treated with OLT to high (50–75%) in patients treated with resection or local ablative techniques. Survival of patients at the early stage treated by hepatic resection is largely conditioned by the degree of portal hypertension and serum levels of bilirubin, which are post-operative predictors of decompensation, but nonetheless also HBV-etiology was shown to influence patient outcomes [160]. An Asiatic study compared the impact of etiology (metabolic and HBV) on survival and recurrence of HCC patients undergoing hepatectomy. HBV impacted negatively on disease-free survival (DFS) and OS at 5-year (39.8 and 49.8%, respectively) compared to Metabolic-HCCs (53 and 63%, respectively), as also confirmed from the 5-years DFS and OS of the Met-HBV-HCC group, where the HBV seemed to give outcomes similar to those of HBV-HCC group (DFS 40.2% and OS 47.5%) [161]. Recurrence within 24 months after resection is defined as “early”, and its predictive factors are microscopic vascular invasion, high pre-treatment levels of serum AFP (alpha-fetoprotein), and non-anatomical resection. “Late” recurrence after resection (after 24 months) is predicted by the grade of hepatitis activity and number of HCC nodules [162]. Among ablation therapies, a single-center study of 249 HBV-related HCCs who underwent RFA or TACE-RFA showed a 5-year OS rate of 58.3% (median OS of 66 months) and 65.46% rate of recurrence during median follow-up of 53 months. Moreover, Child–Pugh class B was identified as an independent prognostic factor for OS among these patients [163].

### 13.2. Outcomes of Liver Transplantation on HBV-Associated HCC

In recent decades, survival after orthotopic liver transplantation (OLT) in HBV-related HCC has remained successfully stable, showing a substantially unvaried 5-year survival over time (approximately 80%). Therefore, in a recent American analysis of the OPTN/UNOS registry, the authors compared survivals of HCV-HCC vs. HBV-HCC in the pre- and post-direct-acting antivirals (DAAs) era. Beyond the improved survival in HCV-related HCC, thanks to the sustained viral response (SVR) after DAAs, HBV-related HCCs undergoing OLT showed a stable survival between the pre-DAAs era (5-year and 10-year survival of 80.5% and 71%, respectively) and the post-DAAs era (5-year survival of 83.4%) [164,165]. These findings, even if encouraging about the efficacy of OLT, also show few improvements in the setting of HBV-HCC undergoing LT. An explanation is the lack of new therapeutic strategies, which may change the natural history of both HBV liver disease and HBV-HCC, just as DAAs did for HCV. Furthermore, HDV coinfection could weigh on HBV liver disease, even worsening the outcomes after OLT for HBV–HDV-related HCC. Therefore, HBV recurrence after LDLT was found to be a risk factor for HCC recurrence, especially in patients with HBV–HDV coinfection, and HBV-HCC co-recurrence was 4.99-fold higher when HDV was present (HDV was an independent risk factor for HBV-HCC co-recurrence in the logistic regression analysis). Those findings are from a single-center study analyzing 355 patients with HCC among 1005 living donor liver transplantations (LDLT) for HBV-related liver disease (including HBV–HDV coinfected patients) [166]. Therefore, HDV coinfection accelerates the progression of HBV-related liver disease, but it is also hypothesized that HBV and HDV co-infection may cause a more aggressive tumor, whose result is the recurrence after LDLT [167].

### 13.3. Efficacy of Systemic Therapies and Their Impact on HBV-Associated HCC

In 2022, oncologists and hepatologists have a bigger armory to treat advanced HCC (aHCC) compared to that available a few years ago. Based on the new insights into systemic therapies for advanced HCC (aHCC), the American and European guidelines [168,169] recently introduced a combination of atezolizumab plus bevacizumab as first-line therapy, preferring this immune-checkpoint inhibitors (ICIs)-based regimen over the TKI (tyrosine-kinase inhibitors)-based first-line regimen (sorafenib, lenvatinib). The first drug available for aHCC was sorafenib, in 2008, after the publication of results from the SHARP trial, where only 19% of patients had HBV-related HCC in the sorafenib arm [170]. Later, the ASIAN-PACIFIC trial [171] explored sorafenib efficacy on Asian HCCs in a cohort including 70.7% of patients with chronic HBV infection, and it showed an OS slightly lower compared to the SHARP trial (median OS 6.5 vs. 10.7 months, respectively), even if other outcomes (PFS, ORR, TTP, and safety profile) followed previous results. Moreover, the REFLECT trial (lenvatinib vs. sorafenib for first-line in aHCC, non-inferiority trial) included half of the patients with HBV-aHCC (53% in the lenvatinib and 48% in the sorafenib arm), showing a similar OS (median 13.6 vs. 12.3 months, respectively) but higher PFS for the lenvatinib arm compared to the sorafenib arm (7.4 vs. 3.7 months, respectively) [172]. Therefore, lenvatinib could be preferred to sorafenib as first-line systemic therapy when patients are affected by HBV-related aHCC, but further prospective clinical trials are needed to confirm this point. Nonetheless, in the last two years, the demonstrated superior efficacy of combination immunotherapy (atezolizumab–bevacizumab) vs. sorafenib (HR 0.58 for OS; *p* < 0.001) permitted improvement in survival of advanced HCC (median OS 19.2 vs. 13.2 for sorafenib), becoming the preferred first-line systemic therapy for every etiology of liver disease [173]. Moreover, another combination immunotherapy for aHCC (durvalumab plus tremelimumab) recently showed better efficacy vs. aorafenib in preliminary results (median OS 16.4 vs. 13.8 for aorafenib) [174]. Therefore, nowadays, the previous considerations regarding the choice among TKIs for first-line in HBV-aHCC were limited to those cases in which an ICI-based regimen is not available in routine clinical practice. Regarding second-line therapies (regorafenib, ramucirumab, cabozantinib), the percentage of HBV patients in the cohorts of registrative trials are similar (approximately among 35 and 38% of HBV patient in every arm of the trials—Table 3), and their choice mainly depends on the previous first-line, patient tolerance, or tumor progression, as indicated in the guidelines [175,176,177]. However, outcomes during HCC systemic treatments are always dependent on the underlying liver function, because these treatments may damage the functioning liver and decompensate liver disease, which can lead to a temporary or permanent treatment discontinuation. In fact, the maintenance of an optimal liver function is fundamental, because it permits the best strategy of HCC treatment to be applied [178].

## 14. Specific Therapies of HBV-Related HCC

In the future, new strategies and treatments are going to be developed, and many hypothetic pathways and proteins, which are under evaluation at the moment, will become new targets. In the contest of HBV-related HCC, chimeric antigen receptor (CAR)- and T cell receptor (TCR)-T cells targeting HBV antigens have shown antiviral and anti-HCC activity in vitro [181,182]. Studies on CAR-T and TCR-T cells under different HBV-associated pathogenic states are ongoing, with preliminary data indicating clinical benefit [183]. CAR-T is a type of treatment in which T cells are taken from a patient’s blood, and then a gene for CAR which binds a certain protein on the patient’s cancer cells is added to the T cells in the laboratory. Tan et al. describes a CAR-T cell technology that can recognize HBV-specific epitopes in HCCs. Since HBV-associated HCCs seem to not contain actively replicating viruses and only express partially integrated/truncated proteins, T-cells can be designed to target these specific tumor-associated antigens. In one of these undergoing studies, one patient treated with HBV-specific CAR-T cells experienced a response with reduction of five out of six pulmonary metastases in one year [184]. Furthermore, the persistence of CAR-T technology in viral-related HCC could be achieved by engineering a separate CAR-T receptor to recognize viral antigens to boost T-cells while targeting cancer-specific lesions. In fact, as supposed in the transcriptome analysis of Lim et al. [91], novel immunotherapeutic or a combination of therapies that target specific pathways in etiology-related HCC could be designed for better disease management when molecular pathways will be clearer in etiology-related HCC. Another future perspective is the application of mucosal-associated invariant T (MAIT) cells in HBV-related HCC patients. MAIT cells are naturally enriched in the liver and represent a critical innate-like T cell subset with a potent intrahepatic effector [185,186]. Healy et al. investigated their TCR redirecting MAIT cells in the context of HBV using a preclinical HCC cell model, with the limit of using healthy donor peripheral blood mononuclear cells (PBMCs) when performing all functional experiments. Despite the limits, their findings support future applications of MAIT cells for liver-directed immunotherapies in HBV-related HCC [187]. Furthermore, a single-clonal origin of HBV-related HCC is also debated due to data based on deep-sequencing studies indicating multifocal HCC as a totality of independent tumors or as intrahepatic metastases, even if confined within a tumor mass [188,189,190]. Whether multinodular HCC derives from multiple carcinogenesis events under chronic hepatitis/cirrhosis, maybe it could explain better outcomes of combination systemic therapies, even if it remains unclear at the moment.

## 15. Conclusions

Persistent viral infection and immune-mediated damage cause significant and complicated changes over time in the liver microenvironment and are undoubtedly risk factors for the development of HCC in those patients with HBV infection. Many different genetic and molecular pathways are involved in the development of HBV-related HCC and are still under investigation. Current treatment options for HBV reduce HCC risk but do not completely eliminate it. Moreover, HDV coinfection increases the risk of HCC, but new treatment options have just been approved, so their efficacy may help to reduce this risk and reduce progression of liver disease in the next years. Regarding future approaches to systemic therapy, evidence of the increased expression of PD-1 on TREG and TRM in HBV-related HCC is congruent with a virus-induced immunosuppressive/exhausted TME, as abovementioned [91]. Therefore, treatments with PD-1 inhibitor may give a survival benefit, even if the results of CheckMate 040 (nivolumab) did not find a significant difference in response rate for patients with HBV-related or non-viral-related HCC [191]. Nonetheless, nowadays, ICIs are the preferred choice in first-line systemic therapy thanks to the results of IMbrave150, in which half of patients had HBV-related HCC. In the wake of these results, many RCTs (i.e., HIMALAYA, ORIENT-32) are evaluating other immunotherapies, trying to find the best regimen [174]. Chinese patients with previously untreated HBV-related HCC (94% of entire cohort) are enrolled in ORIENT-32, which compares sintilimab (PD-1 inhibitor) plus bevacizumab biosimilar (IBI305) vs. sorafenib, showing a significant overall survival, progression-free survival, and safety profile in preliminary analysis [180]. However, if the best sequential first- and second-line systemic treatments of HCC remain elusive [192], the maintenance of optimal liver function is absolutely crucial at any stage [178].

In conclusion, HBV-related HCC is a typical example of how the etiology and the management of underlying liver disease can influence the risk of HCC in terms of adequate modality of surveillance and progression of disease. In fact, the etiologic therapy (e.g., antivirals in the case of HBV-related disease) significantly improves all of the outcomes of liver cirrhosis, without and with HCC, and also in other settings [193]. Scientists and clinicians are extremely interested in knowing how the new therapies for HDV will influence in the future the long-term outcomes of HDV-associated liver disease and HCC. Furthermore, the maintenance of liver function in cirrhotic patients is fundamental for reducing the risk of HCC, improving outcomes, and permitting a proper treatment of HCC itself [178]. Considering all of these points, the hepatologist has unavoidably the central role in the journey of patients with HCC of any etiology, even if particular cases can take advantages from a multidisciplinary approach including surgeon, infectivologist, radiologist, and oncologist.

## Figures and Tables

**Table 1:** Differences regarding surveillance among the major guidelines.

			AASLD [104,106]	EASL [94,103]	APASL [102]
**Modality**	**AFP**		Yes *	No	Yes—biannually (in combination with US)
	**US**	Liver Cirrhosis	Yes—every 6 months	Yes—every 6 months	Yes—every 6 months
		Hepatitis	Yes—every 6 months in high-risk patients	Yes—every 6 months in high risk patients ^§^	Yes—in high-risk patients (timing not specified)
**High-risk patients**		Cirrhotic patients	Cirrhosis HBsAg-positive and also with HBsAg seroclearance	Child–Pugh stage A and B Child–Pugh stage C awaiting liver transplantation	HBV-related
		Non-cirrhotic patients	**With HBsAg seroclearance**: a first degree family member with HCC, or a long duration of infection (>40 years for males and >50 years for females who have been infected with HBV from a young age) **HBsAg-positive adults**: -Asian or black men > 40 years -Asian women > 50 years -Persons with a first-degree family member with a history of HCC -Persons with HDV	-**HBsAg-positive patients**: according to PAGE-B classes for Caucasian subjects, respectively 10–17 and ≥18 score points -**F3 patients**, regardless of etiology may be considered for surveillance based on an individual risk assessment	**Chronic HBV carriers**: -Asian female > 50 years, -Asian males > 40 years, -Africans aged > 20 years, -History of HCC in the family

* Optional, but always in combination with US. ^§^ Patients at low HCC risk left untreated for HBV and without regular six months of surveillance must be reassessed at least yearly to verify progression of HCC risk. APASL = Asian Pacific Association for the Study of the Liver; AASLD = American Association for the Study of Liver Diseases; EASL = European Association for the Study of the Liver; HCC = hepatocellular carcinoma; AFP = alpha-fetoprotein; US = ultrasonography; HBV = hepatitis B virus; HDV = hepatitis D virus.

**Table 2:** Scoring system predictive for hepatocellular carcinoma in HBV patients.

					Derivation Cohort					Validation Cohort				
Score, Year	Predictive Time	Variables	c-Index/ AUC	N of Pts; Ethnicity	Age (Mean ± DS)	Male (%)	Cirrhosis, *n* (%)	NUCs, % (*n*)	c-Index/ AUC	N of Pts; Ethnicity	Age (Mean ± DS)	Male (%)	Cirrhosis, *n* (%)	NUCs, % (*n*)
**Untreated patients (Non-NUCs)**														
**CU-HCC, 2009** [108]	10 years	Age, Albumin (g/L), Bilirubin (µmol/L), HBV-DNA (log copies/mL), Cirrhosis	NA	1005, Asians	48 ± 7	67.8	383 (38.1)	15.1 * (152)	0.76, 0.78	424, Asians	41 ± 13	276 (65)	69 (16)	25 (106)
**GAG-HCC, 2008** [109]	5 and 10 years	Gender, Age, HBV-DNA (log copies/mL), core promoter Mutations, Cirrhosis	0.88, 0.89	820, Asians	40.6	69.9	124 (15.1)	0	-	-	-	-	-	-
**REACH-B, 2011** [110]	3, 5, and 10 years	Gender, ALT (U/L), HBeAg (+/−), HBV DNA level (copies per mL)	NA	3584, Asians	45.7 ± 9.8	NA	0	0	0.811, 0.796, 0.769	1505, Asians	41.9 ± 13.5	NA	277 (18.4)	0
**REACH-B II, 2013** [111]	5, 10, and 15 years	Gender, ALT (U/L), family history of HCC (+/−), HBeAg (+/−), HBV DNA level (copies per mL), HBsAg (+/−), genotype	0.89, 0.85, 0.86	2227, Asians	30–65	NA	0	0	0.84, 0.86, 0.87	1113, Asians	NA	NA	0	0
**LSM-HCC, 2014** [112]	3 and 5 years	LSM (kPa), Age, Albumin (g/L), and HBV DNA level (copies per mL)	0.83, 0.83	1035, Asians	46 ± 12	64	331 (32)	38 * (390)	0.89, 0.83	520, Asians	46 ± 12	64	163 (31)	32 (165)
**RWS-HCC, 2016** [113]	10 years	Gender, Age, Cirrhosis (+/−), AFP level	0.915	538, Asians	56.4 ± 12.1	62.6	80 (14.9)	NA	-	-	-	-	-	-
**D^2^AS, 2017** [114]	3 and 5 years	Gender, Age, HBV DNA level (copies per mL)	0.895, 0.884	971, Asians	42.6 ± 10.6	58.1	0	0	0.889, 0.876	507, Asians	42.2 ± 12.4	55.8	0	0
**HCC-ESC, 2018** [115]	5, 10, and 20 years	Age at ESC (HBeAg seroclearance), male sex, cirrhosis, hypoalbuminemia, HBV DNA level (copies per mL) and ALT	0.95, 0.91, 0.92	723, Asians	32	60.6	NA	59.1 * (427)	-	-	-	-	-	-
**AGED, 2019** [116]	5, 10, 15, and 20 years	Gender, Age, HBeAg (+/−), HBV DNA level (copies per mL)	0.76, 0.76, 0.79, 0.80	628, Asians	NA	NA	0	NA	0.73, 0.74	1663, Asians	NA	NA	0	NA
**Treated patients (NUCs)**														
**mREACH-B,2014** [129,130]	3 and 5 years	Age, gender, LSM (kPa), HBeAg (+/−),	0.805	192, Asians	49	69.8	90 (46,9)	NA	0.828, 0.806	1308, Asians	50	67.5	233 (17.8)	64.8 * (848)
**PAGE-B, 2015** [126]	5 years	Age, gender, platelets	0.82	1325, Caucasians	52 ± 21	923 (70)	269 (20)	100	0.82	490, Caucasian	56 ± 14	76	234 (48)	100
**Modified PAGE-B, 2018** [127]	5 years	Age, gender, albumin (g/dL), platelets	0.82	2001, Asians	50	1282 (64.1)	383 (19.1)	100	0.72	1000, Asians	50	63.1	201 (20.1)	100
**HCC-RESCUE, 2017** [117]	1, 3, and 5 years	Age, Gender, Cirrhosis	0.798, 0.788, 0.768	990, Asians	47.4 ± 10.5	65	389 (39.3)	100	0.817, 0.810, 0.809	1071, Asians	46.6 ± 11.5	63	695 (65)	100
**CAMD** [131,132]	1, 2, and 3 year	Age, Gender, Diabetes, Cirrhosis	0.83, 0.82, 0.82	23851, Asians	47.5	74	6308 (26.4)	100	0.74, 0.75, 0.76	19,321, Asians	52.1	66.05	1371 (7.1)	100
**AASL, 2019** [118]	3 and 5 years	Age, Gender, Albumin, Cirrhosis	0.814, 0.802	944, Asians	50	62.1	371 (39.3)	100	0.850, 0.805	298, Asians	53	58.7	116 (38.9)	100
**REAL-B, 2020** [119]	3, 5, and 10 years	Age, Gender, Alcohol, Diabetes, Cirrhosis, Platelets, AFP	0.83, 0.81, 0.81	5365, Caucasians and Asians	48.4 ± 12.7	69.2	1085 (20.2)	100	0.74, 0.73, 0.74	2683, Caucasians and Asians	48.3 ± 12.5	69.1	592 (22.1)	100
**CAMPAS, 2020** [120]	7 years	Age, Gender, Cirrhosis, Platelets, Albumin, LSM	0.874	1511, Asians	49.7	65.5	602 (39.8)	100	0.847	252, Asians	NA	NA	NA	NA
**APA-B, 2017** [121]	2, 3, and 5 years	Age, Platelets, AFP at month 12	0.877, 0.842, 0.827	883, Asians	50 ± 17	72.7	481 (36.3)	97.3	0.939, 0.892, 0.862	442, Asians	49 ± 18	74.2	164 (37.1)	97.3
**HCC-ESC_AVT_, 2020** [122]	3, 5, and 10 years	Age, Gender, Cirrhosis, ALT, AST, Platelets	0.791, 0.770, 0790	769, Asians	47	59.2	319 (41.5)	100	0.802, 0.774, 0.776	1061, Asians	46	62.5	277 (26.1)	100
**CAGE-B, 2020** [123]	In years 5–12 (after the fifth year from starting NUC)	Age and LSM at year 5, Cirrhosis at baseline	0.814	1427, Caucasians	52.1 ± 13.1	69.5	370 (25.9)	100	-	-	-	-	-	-
**SAGE-B, 2020** [123]	In years 5–12 (after the fifth year from starting NUC)	Age and LSM at year 5	0.809	1427, Caucasians	52.1 ± 13.1	69.5	370 (25.9)	100	-	-	-	-	-	-
**Toronto HCC risk index (THRI), 2017 ^§^** [133]	5 and 10 years	Age, etiology, gender, platelets	0.76 ^§^	2079, Caucasians	53 ± 12.4	1251 (60.1)	2079 (100)	76	0.77 °	1144, Caucasian	51.2 ± 11.6	575 (50.3)	1144 (100)	NA

* Untreated patients started antiviral treatment during follow up. ^§^ HBV patients were only 19% in the derivation cohort and 22.1% in the validation cohort. HBV patients showed higher 5- and 10-year cumulative incidence among etiologies. ^§^ c-statistic was 0.72 in HBV subgroup of derivation cohort. ° c-statistic was confirmed to be 0.77 in the HBV subgroup of validation cohort.

**Table 3:** RCT of registrative systemic therapies for HCC exploring the HBV patients considered in the arm of treatments.

Study Name	Arms	N of Patients	N of HBV Patients (%)	Median OS	Median PFS	ORR (%)
**First-Line Therapies**						
**IMbrave150** [173]	Atezolizumab (PD-L1) + bevacizumab (VEGF)	336	164 (49)	19.2	6.8	27
	Sorafenib (TKI)	165	76 (46)	13.2	4.3	12
**SHARP** [170]	Sorafenib (TKI)	299	56 (19)	10.7	-	2
	Placebo	303	55 (18)	7.9	-	1
**REFLECT** [172]	Lenvatinib (TKI)	478	251 (53)	13.6	7.4	40.6
	Sorafenib (TKI)	476	228 (48)	12.3	3.7	12.4
**Second-line Therapies**						
**RESORCE** [176]	Regorafenib (TKI)	379	143 (38)	10.6	3.1	11
	Placebo	194	73 (38)	7.8	1.5	4
**CELESTIAL** [175]	Cabozantinib (TKI)	470	178 (38)	10.2	5.2	4
	Placebo	237	89 (38)	8	1.9	1
**REACH** [179]	Ramucirumab (VEGFR2)	283	100 (35)	9.2	2.8	7
	Placebo	282	101 (36)	7.6	2.1	<1
**REACH-2** [177]	Ramucirumab (VEGFR2)	197	71 (36)	8.5	2.8	5
	Placebo	95	36 (38)	7.3	1.6	1
**Under Evaluation (as First-Line)**						
**HIMALAYA** [174]	Durvalumab (PD-L1) + tremelimumab (CTLA-4)	393	NA	16.4	3.8	20
	Durvalumab (PD-L1)	389	NA	16.6	3.7	17
	Sorafenib (TKI)	389	NA	13.8	4.1	5.1
**ORIENT-32** [180]	Sintilimab PLUS bevacizumab biosimilar	380	359 (94)	NR	4.6	21
	Sorafenib	191	179 (94)	10.4	2.8	4

## References

[1] Bray F., Ferlay J., Soerjomataram I., Siegel R.L., Torre L.A., Jemal A. (2018). Global cancer statistics 2018: GLOBOCAN estimates of incidence and mortality worldwide for 36 cancers in 185 countries. CA Cancer J. Clin..

[2] De Flora S.C.E., Bonanni P., Ferro A., Vitale F. (2015). Vaccines and Cancer Prevention/Screening Working Groups of the Italian Society of Hygiene, Preventive Medicine and Public Health (SItI). Incidence of infection-associated cancers in Italy and prevention strategies. Epidemiol. Prev..

[3] Garuti F., Neri A., Avanzato F., Gramenzi A., Rampoldi D., Rucci P., Farinati F., Giannini E.G., Piscaglia F., Rapaccini G.L. (2021). The changing scenario of hepatocellular carcinoma in Italy: An update. Liver Int..

[4] Vitale A., Svegliati-Baroni G., Ortolani A., Cucco M., Dalla Riva G.V., Giannini E.G., Piscaglia F., Rapaccini G., Di Marco M., Caturelli E. (2021). Epidemiological trends and trajectories of MAFLD-associated hepatocellular carcinoma 2002–2033: The ITA.LI.CA database. Gut.

[5] Brar G., Greten T.F., Graubard B.I., McNeel T.S., Petrick J.L., McGlynn K.A., Altekruse S.F. (2020). Hepatocellular Carcinoma Survival by Etiology: A SEER-Medicare Database Analysis. Hepatol. Commun..

[6] Petruzziello A. (2018). Epidemiology of Hepatitis B Virus (HBV) and Hepatitis C Virus (HCV) Related Hepatocellular Carcinoma. Open Virol. J..

[7] Wang H., Men P., Xiao Y., Gao P., Lv M., Yuan Q., Chen W., Bai S., Wu J. (2019). Hepatitis B infection in the general population of China: A systematic review and meta-analysis. BMC Infect. Dis..

[8] Park N.H., Chung Y.H., Lee H.S. (2010). Impacts of vaccination on hepatitis B viral infections in Korea over a 25-year period. Intervirology.

[9] Luo Z., Li L., Ruan B. (2012). Impact of the implementation of a vaccination strategy on hepatitis B virus infections in China over a 20-year period. Int. J. Infect. Dis..

[10] Ni Y.H., Chen D.S. (2010). Hepatitis B vaccination in children: The Taiwan experience. Pathol. Biol..

[11] Polaris Observatory Collaborators (2018). Global prevalence, treatment, and prevention of hepatitis B virus infection in 2016: A modelling study. Lancet Gastroenterol. Hepatol..

[12] Stockdale A.J., Kreuels B., Henrion M.Y.R., Giorgi E., Kyomuhangi I., de Martel C., Hutin Y., Geretti A.M. (2020). The global prevalence of hepatitis D virus infection: Systematic review and meta-analysis. J. Hepatol..

[13] Chen H.Y., Shen D.T., Ji D.Z., Han P.C., Zhang W.M., Ma J.F., Chen W.S., Goyal H., Pan S., Xu H.G. (2019). Prevalence and burden of hepatitis D virus infection in the global population: A systematic review and meta-analysis. Gut.

[14] Urban S., Neumann-Haefelin C., Lampertico P. (2021). Hepatitis D virus in 2021: Virology, immunology and new treatment approaches for a difficult-to-treat disease. Gut.

[15] Papatheodoridis G.V., Chan H.L., Hansen B.E., Janssen H.L., Lampertico P. (2015). Risk of hepatocellular carcinoma in chronic hepatitis B: Assessment and modification with current antiviral therapy. J. Hepatol..

[16] Sagnelli E., Sagnelli C., Pisaturo M., Macera M., Coppola N. (2014). Epidemiology of acute and chronic hepatitis B and delta over the last 5 decades in Italy. World J. Gastroenterol..

[17] Stroffolini T., Ciancio A., Furlan C., Vinci M., Niro G.A., Russello M., Colloredo G., Morisco F., Coppola N., Babudieri S. (2021). Chronic hepatitis B virus infection in Italy during the twenty-first century: An updated survey in 2019. Eur. J. Clin. Microbiol. Infect. Dis..

[18] Valery P.C., Laversanne M., Clark P.J., Petrick J.L., McGlynn K.A., Bray F. (2018). Projections of primary liver cancer to 2030 in 30 countries worldwide. Hepatology.

[19] Petrick J.L., Florio A.A., Loomba R., McGlynn K.A. (2020). Have incidence rates of liver cancer peaked in the United States?. Cancer.

[20] McGlynn K.A., Petrick J.L., London W.T. (2015). Global epidemiology of hepatocellular carcinoma: An emphasis on demographic and regional variability. Clin. Liver Dis..

[21] Wranke A., Pinheiro Borzacov L.M., Parana R., Lobato C., Hamid S., Ceausu E., Dalekos G.N., Rizzetto M., Turcanu A., Niro G.A. (2018). Clinical and virological heterogeneity of hepatitis delta in different regions world-wide: The Hepatitis Delta International Network (HDIN). Liver Int..

[22] Plummer M., de Martel C., Vignat J., Ferlay J., Bray F., Franceschi S. (2016). Global burden of cancers attributable to infections in 2012: A synthetic analysis. Lancet Glob. Health.

[23] D’Souza S., Lau K.C., Coffin C.S., Patel T.R. (2020). Molecular mechanisms of viral hepatitis induced hepatocellular carcinoma. World J. Gastroenterol..

[24] Kulik L., El-Serag H.B. (2019). Epidemiology and Management of Hepatocellular Carcinoma. Gastroenterology.

[25] Lin C.L., Kao J.H. (2017). Natural history of acute and chronic hepatitis B: The role of HBV genotypes and mutants. Best Pract. Res. Clin. Gastroenterol..

[26] Nguyen D.H., Ludgate L., Hu J. (2008). Hepatitis B virus-cell interactions and pathogenesis. J. Cell. Physiol..

[27] Seeger C., Mason W.S. (2000). Hepatitis B virus biology. Microbiol. Mol. Biol. Rev..

[28] Yang S., Liu Y., Feng X., Wang X., Wu M., Gong L., Shu B., Lu Q., Dong J. (2021). HBx acts as an oncogene and promotes the invasion and metastasis of hepatocellular carcinoma both in vivo and vitro. Dig Liver Dis..

[29] Zhang Y., Yan Q., Gong L., Xu H., Liu B., Fang X., Yu D., Li L., Wei T., Wang Y. (2021). C-terminal truncated HBx initiates hepatocarcinogenesis by downregulating TXNIP and reprogramming glucose metabolism. Oncogene.

[30] Yan H., Zhong G., Xu G., He W., Jing Z., Gao Z., Huang Y., Qi Y., Peng B., Wang H. (2012). Sodium taurocholate cotransporting polypeptide is a functional receptor for human hepatitis B and D virus. eLife.

[31] Ni Y., Lempp F.A., Mehrle S., Nkongolo S., Kaufman C., Falth M., Stindt J., Koniger C., Nassal M., Kubitz R. (2014). Hepatitis B and D viruses exploit sodium taurocholate co-transporting polypeptide for species-specific entry into hepatocytes. Gastroenterology.

[32] Koniger C., Wingert I., Marsmann M., Rosler C., Beck J., Nassal M. (2014). Involvement of the host DNA-repair enzyme TDP2 in formation of the covalently closed circular DNA persistence reservoir of hepatitis B viruses. Proc. Natl. Acad. Sci. USA.

[33] Qi Y., Gao Z., Xu G., Peng B., Liu C., Yan H., Yao Q., Sun G., Liu Y., Tang D. (2016). DNA Polymerase kappa Is a Key Cellular Factor for the Formation of Covalently Closed Circular DNA of Hepatitis B Virus. PLoS Pathog..

[34] Blondot M.L., Bruss V., Kann M. (2016). Intracellular transport and egress of hepatitis B virus. J. Hepatol..

[35] Yang W., Summers J. (1999). Integration of hepadnavirus DNA in infected liver: Evidence for a linear precursor. J. Virol..

[36] Yang W., Summers J. (1998). Infection of ducklings with virus particles containing linear double-stranded duck hepatitis B virus DNA: Illegitimate replication and reversion. J. Virol..

[37] Tu T., Budzinska M.A., Shackel N.A., Urban S. (2017). HBV DNA Integration: Molecular Mechanisms and Clinical Implications. Viruses.

[38] Ringelhan M., McKeating J.A., Protzer U. (2017). Viral hepatitis and liver cancer. Philos. Trans. R. Soc. Lond. B Biol. Sci..

[39] Seeger C., Mason W.S. (2015). Molecular biology of hepatitis B virus infection. Virology.

[40] Yaginuma K., Kobayashi H., Kobayashi M., Morishima T., Matsuyama K., Koike K. (1987). Multiple integration site of hepatitis B virus DNA in hepatocellular carcinoma and chronic active hepatitis tissues from children. J. Virol..

[41] Yan H., Yang Y., Zhang L., Tang G., Wang Y., Xue G., Zhou W., Sun S. (2015). Characterization of the genotype and integration patterns of hepatitis B virus in early- and late-onset hepatocellular carcinoma. Hepatology.

[42] Dong H., Zhang L., Qian Z., Zhu X., Zhu G., Chen Y., Xie X., Ye Q., Zang J., Ren Z. (2015). Identification of HBV-MLL4 Integration and Its Molecular Basis in Chinese Hepatocellular Carcinoma. PLoS ONE.

[43] Sung W.K., Zheng H., Li S., Chen R., Liu X., Li Y., Lee N.P., Lee W.H., Ariyaratne P.N., Tennakoon C. (2012). Genome-wide survey of recurrent HBV integration in hepatocellular carcinoma. Nat. Genet..

[44] Wang H.C., Wu H.C., Chen C.F., Fausto N., Lei H.Y., Su I.J. (2003). Different types of ground glass hepatocytes in chronic hepatitis B virus infection contain specific pre-S mutants that may induce endoplasmic reticulum stress. Am. J. Pathol..

[45] Chisari F.V., Filippi P., Buras J., McLachlan A., Popper H., Pinkert C.A., Palmiter R.D., Brinster R.L. (1987). Structural and pathological effects of synthesis of hepatitis B virus large envelope polypeptide in transgenic mice. Proc. Natl. Acad. Sci. USA.

[46] Ng K.Y., Chai S., Tong M., Guan X.Y., Lin C.H., Ching Y.P., Xie D., Cheng A.S., Ma S. (2016). C-terminal truncated hepatitis B virus X protein promotes hepatocellular carcinogenesis through induction of cancer and stem cell-like properties. Oncotarget.

[47] Ma J., Sun T., Park S., Shen G., Liu J. (2011). The role of hepatitis B virus X protein is related to its differential intracellular localization. Acta Biochim. Biophys. Sin..

[48] Shlomai A., de Jong Y.P., Rice C.M. (2014). Virus associated malignancies: The role of viral hepatitis in hepatocellular carcinoma. Semin. Cancer Biol..

[49] Murakami S. (2001). Hepatitis B virus X protein: A multifunctional viral regulator. J. Gastroenterol..

[50] Feitelson M.A., Duan L.X. (1997). Hepatitis B virus X antigen in the pathogenesis of chronic infections and the development of hepatocellular carcinoma. Am. J. Pathol..

[51] Cha M.Y., Kim C.M., Park Y.M., Ryu W.S. (2004). Hepatitis B virus X protein is essential for the activation of Wnt/beta-catenin signaling in hepatoma cells. Hepatology.

[52] Hsieh A., Kim H.S., Lim S.O., Yu D.Y., Jung G. (2011). Hepatitis B viral X protein interacts with tumor suppressor adenomatous polyposis coli to activate Wnt/beta-catenin signaling. Cancer Lett..

[53] Yoo Y.G., Oh S.H., Park E.S., Cho H., Lee N., Park H., Kim D.K., Yu D.Y., Seong J.K., Lee M.O. (2003). Hepatitis B virus X protein enhances transcriptional activity of hypoxia-inducible factor-1alpha through activation of mitogen-activated protein kinase pathway. J. Biol. Chem..

[54] Martin-Vilchez S., Lara-Pezzi E., Trapero-Marugan M., Moreno-Otero R., Sanz-Cameno P. (2011). The molecular and pathophysiological implications of hepatitis B X antigen in chronic hepatitis B virus infection. Rev. Med. Virol..

[55] Hanahan D., Weinberg R.A. (2011). Hallmarks of cancer: The next generation. Cell.

[56] Tian Y., Yang W., Song J., Wu Y., Ni B. (2013). Hepatitis B virus X protein-induced aberrant epigenetic modifications contributing to human hepatocellular carcinoma pathogenesis. Mol. Cell Biol..

[57] Jung S.Y., Kim Y.J. (2013). C-terminal region of HBx is crucial for mitochondrial DNA damage. Cancer Lett..

[58] Liang H.W., Wang N., Wang Y., Wang F., Fu Z., Yan X., Zhu H., Diao W., Ding Y., Chen X. (2016). Hepatitis B virus-human chimeric transcript HBx-LINE1 promotes hepatic injury via sequestering cellular microRNA-122. J. Hepatol..

[59] Zhang B., Han S., Feng B., Chu X., Chen L., Wang R. (2017). Hepatitis B virus X protein-mediated non-coding RNA aberrations in the development of human hepatocellular carcinoma. Exp. Mol. Med..

[60] Kitada T., Seki S., Iwai S., Yamada T., Sakaguchi H., Wakasa K. (2001). In situ detection of oxidative DNA damage, 8-hydroxydeoxyguanosine, in chronic human liver disease. J. Hepatol..

[61] Gao W.Y., Li D., Cai D.E., Huang X.Y., Zheng B.Y., Huang Y.H., Chen Z.X., Wang X.Z. (2017). Hepatitis B virus X protein sensitizes HL-7702 cells to oxidative stress-induced apoptosis through modulation of the mitochondrial permeability transition pore. Oncol. Rep..

[62] Xie W.H., Ding J., Xie X.X., Yang X.H., Wu X.F., Chen Z.X., Guo Q.L., Gao W.Y., Wang X.Z., Li D. (2020). Hepatitis B virus X protein promotes liver cell pyroptosis under oxidative stress through NLRP3 inflammasome activation. Inflamm. Res..

[63] Lee Y.I., Hwang J.M., Im J.H., Lee Y.I., Kim N.S., Kim D.G., Yu D.Y., Moon H.B., Park S.K. (2004). Human hepatitis B virus-X protein alters mitochondrial function and physiology in human liver cells. J. Biol. Chem..

[64] Ivanov A.V., Valuev-Elliston V.T., Tyurina D.A., Ivanova O.N., Kochetkov S.N., Bartosch B., Isaguliants M.G. (2017). Oxidative stress, a trigger of hepatitis C and B virus-induced liver carcinogenesis. Oncotarget.

[65] Tong S., Revill P. (2016). Overview of hepatitis B viral replication and genetic variability. J. Hepatol..

[66] Walter P., Ron D. (2011). The unfolded protein response: From stress pathway to homeostatic regulation. Science.

[67] Lee H., Kim H., Lee S.A., Won Y.S., Kim H.I., Inn K.S., Kim B.J. (2015). Upregulation of endoplasmic reticulum stress and reactive oxygen species by naturally occurring mutations in hepatitis B virus core antigen. J. Gen. Virol..

[68] Peiffer K.H., Akhras S., Himmelsbach K., Hassemer M., Finkernagel M., Carra G., Nuebling M., Chudy M., Niekamp H., Glebe D. (2015). Intracellular accumulation of subviral HBsAg particles and diminished Nrf2 activation in HBV genotype G expressing cells lead to an increased ROI level. J. Hepatol..

[69] Jiang Y., Han Q., Zhao H., Zhang J. (2021). The Mechanisms of HBV-Induced Hepatocellular Carcinoma. J. Hepatocell. Carcinoma.

[70] Chayanupatkul M., Omino R., Mittal S., Kramer J.R., Richardson P., Thrift A.P., El-Serag H.B., Kanwal F. (2017). Hepatocellular carcinoma in the absence of cirrhosis in patients with chronic hepatitis B virus infection. J. Hepatol..

[71] An P., Xu J., Yu Y., Winkler C.A. (2018). Host and Viral Genetic Variation in HBV-Related Hepatocellular Carcinoma. Front. Genet..

[72] Liu S., Zhang H., Gu C., Yin J., He Y., Xie J., Cao G. (2009). Associations between hepatitis B virus mutations and the risk of hepatocellular carcinoma: A meta-analysis. J. Natl. Cancer Inst..

[73] Yang Y., Sun J.W., Zhao L.G., Bray F., Xiang Y.B. (2015). Quantitative evaluation of hepatitis B virus mutations and hepatocellular carcinoma risk: A meta-analysis of prospective studies. Chin. J. Cancer Res..

[74] Yeh C.T., Chen T., Hsu C.W., Chen Y.C., Lai M.W., Liang K.H., Chen T.C. (2011). Emergence of the rtA181T/sW172* mutant increased the risk of hepatoma occurrence in patients with lamivudine-resistant chronic hepatitis B. BMC Cancer.

[75] Yang Z., Zhuang L., Lu Y., Xu Q., Tang B., Chen X. (2016). Naturally occurring basal core promoter A1762T/G1764A dual mutations increase the risk of HBV-related hepatocellular carcinoma: A meta-analysis. Oncotarget.

[76] Shi Y., Wang J., Wang Y., Wang A., Guo H., Wei F., Mehta S.R., Espitia S., Smith D.M., Liu L. (2016). A novel mutant 10Ala/Arg together with mutant 144Ser/Arg of hepatitis B virus X protein involved in hepatitis B virus-related hepatocarcinogenesis in HepG2 cell lines. Cancer Lett..

[77] Yin J., Xie J., Zhang H., Shen Q., Han L., Lu W., Han Y., Li C., Ni W., Wang H. (2010). Significant association of different preS mutations with hepatitis B-related cirrhosis or hepatocellular carcinoma. J. Gastroenterol..

[78] Gao Q., Zhu H., Dong L., Shi W., Chen R., Song Z., Huang C., Li J., Dong X., Zhou Y. (2019). Integrated Proteogenomic Characterization of HBV-Related Hepatocellular Carcinoma. Cell.

[79] Totoki Y., Tatsuno K., Covington K.R., Ueda H., Creighton C.J., Kato M., Tsuji S., Donehower L.A., Slagle B.L., Nakamura H. (2014). Trans-ancestry mutational landscape of hepatocellular carcinoma genomes. Nat. Genet..

[80] Trung N.T., Hoan N.X., Trung P.Q., Binh M.T., Van Tong H., Toan N.L., Bang M.H., Song L.H. (2020). Clinical significance of combined circulating TERT promoter mutations and miR-122 expression for screening HBV-related hepatocellular carcinoma. Sci. Rep..

[81] Yao S., Johnson C., Hu Q., Yan L., Liu B., Ambrosone C.B., Wang J., Liu S. (2016). Differences in somatic mutation landscape of hepatocellular carcinoma in Asian American and European American populations. Oncotarget.

[82] Fujimoto A., Totoki Y., Abe T., Boroevich K.A., Hosoda F., Nguyen H.H., Aoki M., Hosono N., Kubo M., Miya F. (2012). Whole-genome sequencing of liver cancers identifies etiological influences on mutation patterns and recurrent mutations in chromatin regulators. Nat. Genet..

[83] Kawai-Kitahata F., Asahina Y., Tanaka S., Kakinuma S., Murakawa M., Nitta S., Watanabe T., Otani S., Taniguchi M., Goto F. (2016). Comprehensive analyses of mutations and hepatitis B virus integration in hepatocellular carcinoma with clinicopathological features. J. Gastroenterol..

[84] Schulze K., Imbeaud S., Letouze E., Alexandrov L.B., Calderaro J., Rebouissou S., Couchy G., Meiller C., Shinde J., Soysouvanh F. (2015). Exome sequencing of hepatocellular carcinomas identifies new mutational signatures and potential therapeutic targets. Nat. Genet..

[85] Nault J.C., Mallet M., Pilati C., Calderaro J., Bioulac-Sage P., Laurent C., Laurent A., Cherqui D., Balabaud C., Zucman-Rossi J. (2013). High frequency of telomerase reverse-transcriptase promoter somatic mutations in hepatocellular carcinoma and preneoplastic lesions. Nat. Commun..

[86] Li M., Zhao H., Zhang X., Wood L.D., Anders R.A., Choti M.A., Pawlik T.M., Daniel H.D., Kannangai R., Offerhaus G.J. (2011). Inactivating mutations of the chromatin remodeling gene ARID2 in hepatocellular carcinoma. Nat. Genet..

[87] Chaudhary B., Elkord E. (2016). Regulatory T Cells in the Tumor Microenvironment and Cancer Progression: Role and Therapeutic Targeting. Vaccines.

[88] Fu J., Xu D., Liu Z., Shi M., Zhao P., Fu B., Zhang Z., Yang H., Zhang H., Zhou C. (2007). Increased regulatory T cells correlate with CD8 T-cell impairment and poor survival in hepatocellular carcinoma patients. Gastroenterology.

[89] Pallett L.J., Davies J., Colbeck E.J., Robertson F., Hansi N., Easom N.J.W., Burton A.R., Stegmann K.A., Schurich A., Swadling L. (2017). IL-2(high) tissue-resident T cells in the human liver: Sentinels for hepatotropic infection. J. Exp. Med..

[90] Shang B., Liu Y., Jiang S.J., Liu Y. (2015). Prognostic value of tumor-infiltrating FoxP3+ regulatory T cells in cancers: A systematic review and meta-analysis. Sci. Rep..

[91] Lim C.J., Lee Y.H., Pan L., Lai L., Chua C., Wasser M., Lim T.K.H., Yeong J., Toh H.C., Lee S.Y. (2019). Multidimensional analyses reveal distinct immune microenvironment in hepatitis B virus-related hepatocellular carcinoma. Gut.

[92] Jia L., Gao Y., He Y., Hooper J.D., Yang P. (2020). HBV induced hepatocellular carcinoma and related potential immunotherapy. Pharmacol. Res..

[93] Indolfi G., Easterbrook P., Dusheiko G., Siberry G., Chang M.H., Thorne C., Bulterys M., Chan P.L., El-Sayed M.H., Giaquinto C. (2019). Hepatitis B virus infection in children and adolescents. Lancet Gastroenterol. Hepatol..

[94] European Association for the Study of the Liver (2017). European Association for the Study of the, EASL 2017 Clinical Practice Guidelines on the management of hepatitis B virus infection. J. Hepatol..

[95] Wang H.C., Huang W., Lai M.D., Su I.J. (2006). Hepatitis B virus pre-S mutants, endoplasmic reticulum stress and hepatocarcinogenesis. Cancer Sci..

[96] Rizzetto M., Verme G., Recchia S., Bonino F., Farci P., Arico S., Calzia R., Picciotto A., Colombo M., Popper H. (1983). Chronic hepatitis in carriers of hepatitis B surface antigen, with intrahepatic expression of the delta antigen. An active and progressive disease unresponsive to immunosuppressive treatment. Ann. Intern. Med..

[97] Baskiran A., Atay A., Baskiran D.Y., Akbulut S. (2021). Hepatitis B/D-Related Hepatocellular Carcinoma. A Clinical Literature Review. J. Gastrointest. Cancer.

[98] Alfaiate D., Clement S., Gomes D., Goossens N., Negro F. (2020). Chronic hepatitis D and hepatocellular carcinoma: A systematic review and meta-analysis of observational studies. J. Hepatol..

[99] Farci P., Niro G.A., Zamboni F., Diaz G. (2021). Hepatitis D Virus and Hepatocellular Carcinoma. Viruses.

[100] Diaz G., Engle R.E., Tice A., Melis M., Montenegro S., Rodriguez-Canales J., Hanson J., Emmert-Buck M.R., Bock K.W., Moore I.N. (2018). Molecular Signature and Mechanisms of Hepatitis D Virus-Associated Hepatocellular Carcinoma. Mol. Cancer Res..

[101] Singal A.G., Zhang E., Narasimman M., Rich N.E., Waljee A.K., Hoshida Y., Yang J.D., Reig M., Cabibbo G., Nahon P. (2022). HCC Surveillance Improves Early Detection, Curative Treatment Receipt, and Survival in Patients with Cirrhosis: A Systematic Review and Meta-Analysis. J. Hepatol..

[102] Omata M., Cheng A.L., Kokudo N., Kudo M., Lee J.M., Jia J., Tateishi R., Han K.H., Chawla Y.K., Shiina S. (2017). Asia-Pacific clinical practice guidelines on the management of hepatocellular carcinoma: A 2017 update. Hepatol. Int..

[103] European Association for the Study of the Liver (2018). EASL Clinical Practice Guidelines: Management of hepatocellular carcinoma. J. Hepatol..

[104] Terrault N.A., Lok A.S.F., McMahon B.J., Chang K.M., Hwang J.P., Jonas M.M., Brown R.S., Jr Bzowej N.H., Wong J.B. (2018). Update on prevention, diagnosis, and treatment of chronic hepatitis B: AASLD 2018 hepatitis B guidance. Hepatology.

[105] Yang J.D., Mohamed E.A., Aziz A.O., Shousha H.I., Hashem M.B., Nabeel M.M., Abdelmaksoud A.H., Elbaz T.M., Afihene M.Y., Duduyemi B.M. (2017). Characteristics, management, and outcomes of patients with hepatocellular carcinoma in Africa: A multicountry observational study from the Africa Liver Cancer Consortium. Lancet Gastroenterol. Hepatol..

[106] Heimbach J.K., Kulik L.M., Finn R.S., Sirlin C.B., Abecassis M.M., Roberts L.R., Zhu A.X., Murad M.H., Marrero J.A. (2018). AASLD guidelines for the treatment of hepatocellular carcinoma. Hepatology.

[107] Guo J., Gao X.S. (2021). Prediction models for development of hepatocellular carcinoma in chronic hepatitis B patients. World J. Clin. Cases.

[108] Wong V.W., Chan S.L., Mo F., Chan T.C., Loong H.H., Wong G.L., Lui Y.Y., Chan A.T., Sung J.J., Yeo W. (2010). Clinical scoring system to predict hepatocellular carcinoma in chronic hepatitis B carriers. J. Clin. Oncol..

[109] Yuen M.F., Tanaka Y., Fong D.Y., Fung J., Wong D.K., Yuen J.C., But D.Y., Chan A.O., Wong B.C., Mizokami M. (2009). Independent risk factors and predictive score for the development of hepatocellular carcinoma in chronic hepatitis B. J. Hepatol..

[110] Yang H.I., Yuen M.F., Chan H.L., Han K.H., Chen P.J., Kim D.Y., Ahn S.H., Chen C.J., Wong V.W., Seto W.K. (2011). Risk estimation for hepatocellular carcinoma in chronic hepatitis B (REACH-B): Development and validation of a predictive score. Lancet Oncol..

[111] Lee M.H., Yang H.I., Liu J., Batrla-Utermann R., Jen C.L., Iloeje U.H., Lu S.N., You S.L., Wang L.Y., Chen C.J. (2013). Prediction models of long-term cirrhosis and hepatocellular carcinoma risk in chronic hepatitis B patients: Risk scores integrating host and virus profiles. Hepatology.

[112] Wong G.L., Chan H.L., Wong C.K., Leung C., Chan C.Y., Ho P.P., Chung V.C., Chan Z.C., Tse Y.K., Chim A.M. (2014). Liver stiffness-based optimization of hepatocellular carcinoma risk score in patients with chronic hepatitis B. J. Hepatol..

[113] Poh Z., Shen L., Yang H.I., Seto W.K., Wong V.W., Lin C.Y., Goh B.B., Chang P.E., Chan H.L., Yuen M.F. (2016). Real-world risk score for hepatocellular carcinoma (RWS-HCC): A clinically practical risk predictor for HCC in chronic hepatitis B. Gut.

[114] Sinn D.H., Lee J.H., Kim K., Ahn J.H., Lee J.H., Kim J.H., Lee D.H., Yoon J.H., Kang W., Gwak G.Y. (2017). A Novel Model for Predicting Hepatocellular Carcinoma Development in Patients with Chronic Hepatitis B and Normal Alanine Aminotransferase Levels. Gut Liver.

[115] Fung J., Cheung K.S., Wong D.K., Mak L.Y., To W.P., Seto W.K., Lai C.L., Yuen M.F. (2018). Long-term outcomes and predictive scores for hepatocellular carcinoma and hepatitis B surface antigen seroclearance after hepatitis B e-antigen seroclearance. Hepatology.

[116] Fan C., Li M., Gan Y., Chen T., Sun Y., Lu J., Wang J., Jin Y., Lu J., Qian G. (2019). A simple AGED score for risk classification of primary liver cancer: Development and validation with long-term prospective HBsAg-positive cohorts in Qidong, China. Gut.

[117] Sohn W., Cho J.Y., Kim J.H., Lee J.I., Kim H.J., Woo M.A., Jung S.H., Paik Y.H. (2017). Risk score model for the development of hepatocellular carcinoma in treatment-naive patients receiving oral antiviral treatment for chronic hepatitis B. Clin. Mol. Hepatol..

[118] Yu J.H., Suh Y.J., Jin Y.J., Heo N.Y., Jang J.W., You C.R., An H.Y., Lee J.W. (2019). Prediction model for hepatocellular carcinoma risk in treatment-naive chronic hepatitis B patients receiving entecavir/tenofovir. Eur. J. Gastroenterol. Hepatol..

[119] Yang H.I., Yeh M.L., Wong G.L., Peng C.Y., Chen C.H., Trinh H.N., Cheung K.S., Xie Q., Su T.H., Kozuka R. (2020). Real-World Effectiveness from the Asia Pacific Rim Liver Consortium for HBV Risk Score for the Prediction of Hepatocellular Carcinoma in Chronic Hepatitis B Patients Treated with Oral Antiviral Therapy. J. Infect. Dis..

[120] Lee H.W., Park S.Y., Lee M., Lee E.J., Lee J., Kim S.U., Park J.Y., Kim D.Y., Ahn S.H., Kim B.K. (2020). An optimized hepatocellular carcinoma prediction model for chronic hepatitis B with well-controlled viremia. Liver Int..

[121] Chen C.H., Lee C.M., Lai H.C., Hu T.H., Su W.P., Lu S.N., Lin C.H., Hung C.H., Wang J.H., Lee M.H. (2017). Prediction model of hepatocellular carcinoma risk in Asian patients with chronic hepatitis B treated with entecavir. Oncotarget.

[122] Lim T.S., Lee H.W., Lee J.I., Kim I.H., Lee C.H., Jang B.K., Chung W.J., Yim H.J., Suh S.J., Seo Y.S. (2020). Predictive score for hepatocellular carcinoma after hepatitis B e antigen loss in patients treated with entecavir or tenofovir. J. Viral Hepat..

[123] Papatheodoridis G.V., Sypsa V., Dalekos G.N., Yurdaydin C., Van Boemmel F., Buti M., Calleja J.L., Chi H., Goulis J., Manolakopoulos S. (2020). Hepatocellular carcinoma prediction beyond year 5 of oral therapy in a large cohort of Caucasian patients with chronic hepatitis B. J. Hepatol..

[124] Hsu Y.C., Ho H.J., Lee T.Y., Huang Y.T., Wu M.S., Lin J.T., Wu C.Y., El-Serag H.B. (2018). Temporal trend and risk determinants of hepatocellular carcinoma in chronic hepatitis B patients on entecavir or tenofovir. J. Viral Hepat..

[125] Kim M.N., Hwang S.G., Rim K.S., Kim B.K., Park J.Y., Kim D.Y., Ahn S.H., Han K.H., Kim S.U. (2017). Validation of PAGE-B model in Asian chronic hepatitis B patients receiving entecavir or tenofovir. Liver Int..

[126] Papatheodoridis G., Dalekos G., Sypsa V., Yurdaydin C., Buti M., Goulis J., Calleja J.L., Chi H., Manolakopoulos S., Mangia G. (2016). PAGE-B predicts the risk of developing hepatocellular carcinoma in Caucasians with chronic hepatitis B on 5-year antiviral therapy. J. Hepatol..

[127] Kim J.H., Kim Y.D., Lee M., Jun B.G., Kim T.S., Suk K.T., Kang S.H., Kim M.Y., Cheon G.J., Kim D.J. (2018). Modified PAGE-B score predicts the risk of hepatocellular carcinoma in Asians with chronic hepatitis B on antiviral therapy. J. Hepatol..

[128] Papatheodoridis G.V., Idilman R., Dalekos G.N., Buti M., Chi H., van Boemmel F., Calleja J.L., Sypsa V., Goulis J., Manolakopoulos S. (2017). The risk of hepatocellular carcinoma decreases after the first 5 years of entecavir or tenofovir in Caucasians with chronic hepatitis B. Hepatology.

[129] Lee H.W., Yoo E.J., Kim B.K., Kim S.U., Park J.Y., Kim D.Y., Ahn S.H., Han K.H. (2014). Prediction of development of liver-related events by transient elastography in hepatitis B patients with complete virological response on antiviral therapy. Am. J. Gastroenterol..

[130] Jung K.S., Kim S.U., Song K., Park J.Y., Kim D.Y., Ahn S.H., Kim B.K., Han K.H. (2015). Validation of hepatitis B virus-related hepatocellular carcinoma prediction models in the era of antiviral therapy. Hepatology.

[131] Hsu Y.C., Yip T.C., Ho H.J., Wong V.W., Huang Y.T., El-Serag H.B., Lee T.Y., Wu M.S., Lin J.T., Wong G.L. (2018). Development of a scoring system to predict hepatocellular carcinoma in Asians on antivirals for chronic hepatitis B. J. Hepatol..

[132] Kim S.U., Seo Y.S., Lee H.A., Kim M.N., Kim E.H., Kim H.Y., Lee Y.R., Lee H.W., Park J.Y., Kim D.Y. (2020). Validation of the CAMD Score in Patients with Chronic Hepatitis B Virus Infection Receiving Antiviral Therapy. Clin. Gastroenterol. Hepatol..

[133] Sharma S.A., Kowgier M., Hansen B.E., Brouwer W.P., Maan R., Wong D., Shah H., Khalili K., Yim C., Heathcote E.J. (2018). Toronto HCC risk index: A validated scoring system to predict 10-year risk of HCC in patients with cirrhosis. J. Hepatol..

[134] Lo A.O., Wong G.L. (2014). Current developments in nucleoside/nucleotide analogues for hepatitis B. Expert Rev. Gastroenterol. Hepatol..

[135] Schutte K., Balbisi F., Malfertheiner P. (2016). Prevention of Hepatocellular Carcinoma. Gastrointest. Tumors.

[136] Stadler D., Kachele M., Jones A.N., Hess J., Urban C., Schneider J., Xia Y., Oswald A., Nebioglu F., Bester R. (2021). Interferon-induced degradation of the persistent hepatitis B virus cccDNA form depends on ISG20. EMBO Rep..

[137] Revill P.A., Tu T., Netter H.J., Yuen L.K.W., Locarnini S.A., Littlejohn M. (2020). The evolution and clinical impact of hepatitis B virus genome diversity. Nat. Rev. Gastroenterol. Hepatol..

[138] Tu T., Zehnder B., Qu B., Ni Y., Main N., Allweiss L., Dandri M., Shackel N., George J., Urban S. (2020). A novel method to precisely quantify hepatitis B virus covalently closed circular (ccc)DNA formation and maintenance. Antivir. Res..

[139] Liu K., Choi J., Le A., Yip T.C., Wong V.W., Chan S.L., Chan H.L., Nguyen M.H., Lim Y.S., Wong G.L. (2019). Tenofovir disoproxil fumarate reduces hepatocellular carcinoma, decompensation and death in chronic hepatitis B patients with cirrhosis. Aliment. Pharmacol. Ther..

[140] Wong G.L., Chan H.L., Mak C.W., Lee S.K., Ip Z.M., Lam A.T., Iu H.W., Leung J.M., Lai J.W., Lo A.O. (2013). Entecavir treatment reduces hepatic events and deaths in chronic hepatitis B patients with liver cirrhosis. Hepatology.

[141] Chaturvedi V.K., Singh A., Dubey S.K., Hetta H.F., John J., Singh M.P. (2019). Molecular mechanistic insight of hepatitis B virus mediated hepatocellular carcinoma. Microb. Pathog..

[142] Kim J.H., Sinn D.H., Kang W., Gwak G.Y., Paik Y.H., Choi M.S., Lee J.H., Koh K.C., Paik S.W. (2017). Low-level viremia and the increased risk of hepatocellular carcinoma in patients receiving entecavir treatment. Hepatology.

[143] Colombo M., Iavarone M. (2014). Role of antiviral treatment for HCC prevention. Best Pract. Res. Clin. Gastroenterol..

[144] Battistella S., Lynch E.N., Gambato M., Zanetto A., Pellone M., Shalaby S., Sciarrone S.S., Ferrarese A., Germani G., Senzolo M. (2021). Hepatocellular carcinoma risk in patients with HBV-related liver disease receiving antiviral therapy. Minerva Gastroenterol..

[145] Murata K., Asano M., Matsumoto A., Sugiyama M., Nishida N., Tanaka E., Inoue T., Sakamoto M., Enomoto N., Shirasaki T. (2018). Induction of IFN-lambda3 as an additional effect of nucleotide, not nucleoside, analogues: A new potential target for HBV infection. Gut.

[146] Abushahba W., Balan M., Castaneda I., Yuan Y., Reuhl K., Raveche E., de la Torre A., Lasfar A., Kotenko S.V. (2010). Antitumor activity of type I and type III interferons in BNL hepatoma model. Cancer Immunol. Immunother..

[147] Ahn J., Lim J.K., Lee H.M., Lok A.S., Nguyen M., Pan C.Q., Mannalithara A., Te H., Reddy K.R., Trinh H. (2016). Lower Observed Hepatocellular Carcinoma Incidence in Chronic Hepatitis B Patients Treated with Entecavir: Results of the ENUMERATE Study. Am. J. Gastroenterol..

[148] Choi J., Jo C., Lim Y.S. (2021). Tenofovir Versus Entecavir on Recurrence of Hepatitis B Virus-Related Hepatocellular Carcinoma After Surgical Resection. Hepatology.

[149] Choi W.M., Choi J., Lim Y.S. (2021). Effects of Tenofovir vs. Entecavir on Risk of Hepatocellular Carcinoma in Patients with Chronic HBV Infection: A Systematic Review and Meta-analysis. Clin. Gastroenterol. Hepatol..

[150] Hsu Y.C., Wu C.Y., Lane H.Y., Chang C.Y., Tai C.M., Tseng C.H., Lo G.H., Perng D.S., Lin J.T., Mo L.R. (2014). Determinants of hepatocellular carcinoma in cirrhotic patients treated with nucleos(t)ide analogues for chronic hepatitis B. J. Antimicrob. Chemother..

[151] Hirode G., Choi H.S.J., Chen C.H., Su T.H., Seto W.K., Van Hees S., Papatheodoridi M., Lens S., Wong G., Brakenhoff S.M. (2022). Off-Therapy Response After Nucleos(t)ide Analogue Withdrawal in Patients with Chronic Hepatitis B: An International, Multicenter, Multiethnic Cohort (RETRACT-B Study). Gastroenterology.

[152] Asif B., Koh C. (2021). Hepatitis D virus (HDV): Investigational therapeutic agents in clinical trials. Expert Opin. Investig. Drugs.

[153] Kang C., Syed Y.Y. (2020). Bulevirtide: First Approval. Drugs.

[154] Bazinet M., Pantea V., Cebotarescu V., Cojuhari L., Jimbei P., Anderson M., Gersch J., Holzmayer V., Elsner C., Krawczyk A. (2021). Persistent Control of Hepatitis B Virus and Hepatitis Delta Virus Infection Following REP 2139-Ca and Pegylated Interferon Therapy in Chronic Hepatitis B Virus/Hepatitis Delta Virus Coinfection. Hepatol. Commun..

[155] Yurdaydin C., Keskin O., Yurdcu E., Caliskan A., Onem S., Karakaya F., Kalkan C., Karatayli E., Karatayli S., Choong I. (2021). A phase 2 dose-finding study of lonafarnib and ritonavir with or without interferon alpha for chronic delta hepatitis. Hepatology.

[156] Llovet J.M., Bru C., Bruix J. (1999). Prognosis of hepatocellular carcinoma: The BCLC staging classification. Semin. Liver Dis..

[157] Reig M., Forner A., Rimola J., Ferrer-Fabrega J., Burrel M., Garcia-Criado A., Kelley R.K., Galle P.R., Mazzaferro V., Salem R. (2022). BCLC strategy for prognosis prediction and treatment recommendation: The 2022 update. J. Hepatol..

[158] Richani M., Kolly P., Knoepfli M., Herrmann E., Zweifel M., von Tengg-Kobligk H., Candinas D., Dufour J.F. (2016). Treatment allocation in hepatocellular carcinoma: Assessment of the BCLC algorithm. Ann. Hepatol..

[159] Cappelli A., Cucchetti A., Cabibbo G., Mosconi C., Maida M., Attardo S., Pettinari I., Pinna A.D., Golfieri R. (2016). Refining prognosis after trans-arterial chemo-embolization for hepatocellular carcinoma. Liver Int..

[160] Bruix J., Castells A., Bosch J., Feu F., Fuster J., Garcia-Pagan J.C., Visa J., Bru C., Rodes J. (1996). Surgical resection of hepatocellular carcinoma in cirrhotic patients: Prognostic value of preoperative portal pressure. Gastroenterology.

[161] Tian Y., Lyu H., He Y., Xia Y., Li J., Shen F. (2018). Comparison of Hepatectomy for Patients with Metabolic Syndrome-Related HCC and HBV-Related HCC. J. Gastrointest. Surg..

[162] Imamura H., Matsuyama Y., Tanaka E., Ohkubo T., Hasegawa K., Miyagawa S., Sugawara Y., Minagawa M., Takayama T., Kawasaki S. (2003). Risk factors contributing to early and late phase intrahepatic recurrence of hepatocellular carcinoma after hepatectomy. J. Hepatol..

[163] Huang J., Huang W., Guo Y., Cai M., Zhou J., Lin L., Zhu K. (2021). Risk Factors, Patterns, and Long-Term Survival of Recurrence After Radiofrequency Ablation with or Without Transarterial Chemoembolization for Hepatocellular Carcinoma. Front. Oncol..

[164] Tabrizian P., Saberi B., Holzner M.L., Rocha C., Kyung Jung Y., Myers B., Florman S.S., Schwartz M.E. (2021). Outcomes of transplantation for HBV- vs. HCV-related HCC: Impact of DAA HCV therapy in a national analysis of >20,000 patients. HPB.

[165] Guarino M., Sessa A., Cossiga V., Morando F., Caporaso N., Morisco F., Special Interest Group on “Hepatocellular carcinoma and new anti-HCV therapies” of the Italian Association for the Study of the Liver (2018). Direct-acting antivirals and hepatocellular carcinoma in chronic hepatitis C: A few lights and many shadows. World J. Gastroenterol..

[166] Baskiran A., Akbulut S., Sahin T.T., Koc C., Karakas S., Ince V., Yurdaydin C., Yilmaz S. (2020). Effect of HBV-HDV co-infection on HBV-HCC co-recurrence in patients undergoing living donor liver transplantation. Hepatol. Int..

[167] Fattovich G., Giustina G., Christensen E., Pantalena M., Zagni I., Realdi G., Schalm S.W. (2000). Influence of hepatitis delta virus infection on morbidity and mortality in compensated cirrhosis type B. The European Concerted Action on Viral Hepatitis (Eurohep). Gut.

[168] Vogel A., Martinelli E., ESMO Guidelines Committee (2021). Updated treatment recommendations for hepatocellular carcinoma (HCC) from the ESMO Clinical Practice Guidelines. Ann. Oncol..

[169] Gordan J.D., Kennedy E.B., Abou-Alfa G.K., Beg M.S., Brower S.T., Gade T.P., Goff L., Gupta S., Guy J., Harris W.P. (2020). Systemic Therapy for Advanced Hepatocellular Carcinoma: ASCO Guideline. J. Clin. Oncol..

[170] Llovet J.M., Ricci S., Mazzaferro V., Hilgard P., Gane E., Blanc J.F., de Oliveira A.C., Santoro A., Raoul J.L., Forner A. (2008). Sorafenib in advanced hepatocellular carcinoma. N. Engl. J. Med..

[171] Cheng A.L., Kang Y.K., Chen Z., Tsao C.J., Qin S., Kim J.S., Luo R., Feng J., Ye S., Yang T.S. (2009). Efficacy and safety of sorafenib in patients in the Asia-Pacific region with advanced hepatocellular carcinoma: A phase III randomised, double-blind, placebo-controlled trial. Lancet Oncol..

[172] Kudo M., Finn R.S., Qin S., Han K.H., Ikeda K., Piscaglia F., Baron A., Park J.W., Han G., Jassem J. (2018). Lenvatinib versus sorafenib in first-line treatment of patients with unresectable hepatocellular carcinoma: A randomised phase 3 non-inferiority trial. Lancet.

[173] Finn R.S., Qin S., Ikeda M., Galle P.R., Ducreux M., Kim T.Y., Kudo M., Breder V., Merle P., Kaseb A.O. (2020). Atezolizumab plus Bevacizumab in Unresectable Hepatocellular Carcinoma. N. Engl. J. Med..

[174] Abou-Alfa G.K., Chan S.L., Kudo M., Lau G., Kelley R.K., Furuse J., Sukeepaisarnjaroen W., Kang Y.-K., Dao T.V., Toni E.N.D. (2022). Phase 3 randomized, open-label, multicenter study of tremelimumab (T) and durvalumab (D) as first-line therapy in patients (pts) with unresectable hepatocellular carcinoma (uHCC): HIMALAYA. J. Clin. Oncol..

[175] Abou-Alfa G.K., Meyer T., Cheng A.L., El-Khoueiry A.B., Rimassa L., Ryoo B.Y., Cicin I., Merle P., Chen Y., Park J.W. (2018). Cabozantinib in Patients with Advanced and Progressing Hepatocellular Carcinoma. N. Engl. J. Med..

[176] Bruix J., Qin S., Merle P., Granito A., Huang Y.H., Bodoky G., Pracht M., Yokosuka O., Rosmorduc O., Breder V. (2017). Regorafenib for patients with hepatocellular carcinoma who progressed on sorafenib treatment (RESORCE): A randomised, double-blind, placebo-controlled, phase 3 trial. Lancet.

[177] Zhu A.X., Kang Y.K., Yen C.J., Finn R.S., Galle P.R., Llovet J.M., Assenat E., Brandi G., Pracht M., Lim H.Y. (2019). Ramucirumab after sorafenib in patients with advanced hepatocellular carcinoma and increased alpha-fetoprotein concentrations (REACH-2): A randomised, double-blind, placebo-controlled, phase 3 trial. Lancet Oncol..

[178] Cabibbo G., Aghemo A., Lai Q., Masarone M., Montagnese S., Ponziani F.R., Italian Association for the Study of the Liver (2022). Optimizing systemic therapy for advanced hepatocellular carcinoma: The key role of liver function. Dig Liver Dis..

[179] Zhu A.X., Park J.O., Ryoo B.Y., Yen C.J., Poon R., Pastorelli D., Blanc J.F., Chung H.C., Baron A.D., Pfiffer T.E. (2015). Ramucirumab versus placebo as second-line treatment in patients with advanced hepatocellular carcinoma following first-line therapy with sorafenib (REACH): A randomised, double-blind, multicentre, phase 3 trial. Lancet Oncol..

[180] Ren Z., Xu J., Bai Y., Xu A., Cang S., Du C., Li Q., Lu Y., Chen Y., Guo Y. (2021). Sintilimab plus a bevacizumab biosimilar (IBI305) versus sorafenib in unresectable hepatocellular carcinoma (ORIENT-32): A randomised, open-label, phase 2-3 study. Lancet Oncol..

[181] Gehring A.J., Xue S.A., Ho Z.Z., Teoh D., Ruedl C., Chia A., Koh S., Lim S.G., Maini M.K., Stauss H. (2011). Engineering virus-specific T cells that target HBV infected hepatocytes and hepatocellular carcinoma cell lines. J. Hepatol..

[182] Bohne F., Chmielewski M., Ebert G., Wiegmann K., Kurschner T., Schulze A., Urban S., Kronke M., Abken H., Protzer U. (2008). T cells redirected against hepatitis B virus surface proteins eliminate infected hepatocytes. Gastroenterology.

[183] Qasim W., Brunetto M., Gehring A.J., Xue S.A., Schurich A., Khakpoor A., Zhan H., Ciccorossi P., Gilmour K., Cavallone D. (2015). Immunotherapy of HCC metastases with autologous T cell receptor redirected T cells, targeting HBsAg in a liver transplant patient. J. Hepatol..

[184] Tan A.T., Yang N., Lee Krishnamoorthy T., Oei V., Chua A., Zhao X., Tan H.S., Chia A., Le Bert N., Low D. (2019). Use of Expression Profiles of HBV-DNA Integrated into Genomes of Hepatocellular Carcinoma Cells to Select T Cells for Immunotherapy. Gastroenterology.

[185] Dusseaux M., Martin E., Serriari N., Peguillet I., Premel V., Louis D., Milder M., Le Bourhis L., Soudais C., Treiner E. (2011). Human MAIT cells are xenobiotic-resistant, tissue-targeted, CD161hi IL-17-secreting T cells. Blood.

[186] Jeffery H.C., van Wilgenburg B., Kurioka A., Parekh K., Stirling K., Roberts S., Dutton E.E., Hunter S., Geh D., Braitch M.K. (2016). Biliary epithelium and liver B cells exposed to bacteria activate intrahepatic MAIT cells through MR1. J. Hepatol..

[187] Healy K., Pavesi A., Parrot T., Sobkowiak M.J., Reinsbach S.E., Davanian H., Tan A.T., Aleman S., Sandberg J.K., Bertoletti A. (2021). Human MAIT cells endowed with HBV specificity are cytotoxic and migrate towards HBV-HCC while retaining antimicrobial functions. JHEP Rep..

[188] Xue R., Li R., Guo H., Guo L., Su Z., Ni X., Qi L., Zhang T., Li Q., Zhang Z. (2016). Variable Intra-Tumor Genomic Heterogeneity of Multiple Lesions in Patients with Hepatocellular Carcinoma. Gastroenterology.

[189] Shi J.Y., Xing Q., Duan M., Wang Z.C., Yang L.X., Zhao Y.J., Wang X.Y., Liu Y., Deng M., Ding Z.B. (2016). Inferring the progression of multifocal liver cancer from spatial and temporal genomic heterogeneity. Oncotarget.

[190] Gao Q., Wang X.Y., Zhou J., Fan J. (2015). Multiple carcinogenesis contributes to the heterogeneity of HCC. Nat. Rev. Gastroenterol. Hepatol..

[191] El-Khoueiry A.B., Sangro B., Yau T., Crocenzi T.S., Kudo M., Hsu C., Kim T.Y., Choo S.P., Trojan J., Welling T.H.R. (2017). Nivolumab in patients with advanced hepatocellular carcinoma (CheckMate 040): An open-label, non-comparative, phase 1/2 dose escalation and expansion trial. Lancet.

[192] Cabibbo G., Reig M., Celsa C., Torres F., Battaglia S., Enea M., Rizzo G.E.M., Petta S., Calvaruso V., Di Marco V. (2022). First-Line Immune Checkpoint Inhibitor-Based Sequential Therapies for Advanced Hepatocellular Carcinoma: Rationale for Future Trials. Liver Cancer.

[193] Reig M., Cabibbo G. (2021). Antiviral therapy in the palliative setting of HCC (BCLC-B and -C). J. Hepatol..

